# Supplementary material for: Social clustering of preference for female genital mutilation/cutting in south-central Ethiopia
Source: Nat Hum Behav. 2025 Jun 10;9(9):1802–14. doi: 10.1038/s41562-025-02236-z (PMC12454122; doi:10.1038/s41562-025-02236-z)
Supplement: Supplementary file 1 — Supplementary Methods, Tables 1–33, Figs. 1–14 and References. [file 41562_2025_2236_MOESM1_ESM.pdf]

nature human behaviour

Supplementary information

https://doi.org/10.1038/s41562-025-02236-z

Social clustering of preference for female genital mutilation/cutting in south-central Ethiopia

In the format provided by the authors and unedited

Contents

Methodology – additional details ... 4

Data ... 4

Measures ... 4

Analytical strategy ... 5

Sample characteristics ... 6

Tables ... 7

Table S1 Demographic characteristics by kebele-zone ... 7

Table S2 Empirical expectations across kebele-zones ... 8

Table S3 Normative expectations across kebele-zones ... 9

Table S4 Subsample comparisons ... 10

Table S5 Measures of network centrality by relationship type dependent on FGMC-preference ... 13

Table S6 Kebele-zone-level network characteristics and pro-FGMC preference prevalence ... 14

Table S7 Latent *money-borrowing* network characteristics ... 16

Table S8 Full results from the ALAAM models exploring contagion, i.e. *social influence*, in the estimated *chatting* networks ... 17

Table S9 Full results from the ALAAM models exploring contagion, i.e. *social influence*, in the estimated *respect* networks ... 19

Table S10 Full results from the ALAAM models exploring contagion, i.e. *social influence*, in the latent *money-borrowing* networks ... 21

Table S11 Goodness-of-fit results for models exploring *social influence* in the all alters *chatting* networks (the best fitting model is indicated in bold) ... 23

Table S12 Goodness-of-fit results for models exploring *social influence* in the kin only *chatting* networks (the best fitting model is indicated in bold) ... 24

Table S13 Goodness-of-fit results for models exploring *social influence* in the non-kin only *chatting* networks (the best fitting model is indicated in bold) ... 25

Table S14 Goodness-of-fit results for models exploring *social influence* in the all alters *respect* networks (the best fitting model is indicated in bold) ... 26

Table S15 Goodness-of-fit results for models exploring *social influence* in the kin only *respect* networks (the best fitting model is indicated in bold) ... 27

Table S16 Goodness-of-fit results for models exploring *social influence* in the non-kin only *respect* networks (the best fitting model is indicated in bold) ... 29

Table S17 Goodness-of-fit results for models exploring *social influence* in the all alters *money* networks (full sample, the best fitting model is indicated in bold) ... 31

Table S18 Goodness-of-fit results for models exploring *social influence* in the kin only *money* networks (full sample, the best fitting model is indicated in bold) ... 32

Table S19 Goodness-of-fit results for models exploring *social influence* in the non-kin only *money* networks (full sample, the best fitting model is indicated in bold) 33

Table S20 Full results from the best fitting ALAAM models exploring contagion, i.e. *social influence*, in the estimated kin and non-kin only *chatting* networks 35

Table S21 Full results from the best fitting ALAAM models exploring contagion, i.e. *social influence*, in the estimated *respect* networks 36

Table S22 Full results from the best fitting ALAAM models exploring contagion, i.e. *social influence*, in the estimated *money* networks (full sample) 37

Table S23 Full results from the STRAND kebele-zone 1 model exploring *social selection* in the latent *marriage-advice* networks 38

Table S24 Full results from the STRAND kebele-zone 2 model exploring *social selection* in the latent *marriage-advice* networks 39

Table S25 Full results from the STRAND kebele-zone 3 model exploring *social selection* in the latent *marriage-advice* networks 40

Table S26 Full results from the STRAND kebele-zone 4 model exploring *social selection* in the latent *marriage-advice* networks 41

Table S27 Full results from the STRAND kebele-zone 5 model exploring *social selection* in the latent *marriage-advice* networks 42

Table S28 Full results from the STRAND kebele-zone 6 model exploring *social selection* in the latent *marriage-advice* networks 43

Table S29 Full results from the STRAND kebele-zone 7 model exploring *social selection* in the latent *marriage-advice* networks 44

Table S30 Full results from the STRAND kebele-zone 8 model exploring *social selection* in the latent *marriage-advice* networks 45

Table S31 Full results from the STRAND kebele-zone 9 model exploring *social selection* in the latent *marriage-advice* networks 46

Table S32 Demographic characteristics by kebele-zone for the full sample and the network respondents subsample 47

Table S33 Within-kebele-zone advice out and advice in nominations used in the STRAND models (n = number of nominators) 48

Figures 49

Figure S1 Directed acyclic graph (DAG) 49

Figure S2 The distribution of FGMC preference dependent on A) age, B) gender, C) highest education level, and D) community role 50

Figure S3 Comparing empirical expectations between the two largest kebele-zones 51

Table S4 The proportional distribution of relationship types among network ties by FGMC preference 52

Figure S5 Plot of *respect* ties 53

Figure S6 Plot of estimated latent *money-borrowing* ties 54

Figure S7 Plot of estimated latent *marriage-advice* ties 55

Figure S8 Social influence signals across networks. Plotted are the posterior distributions for the estimates for social contagion from the best fitting ALAAM models assessing either all, kin-only, or non-kin-only alters within the A) *chatting,* B) *respect*, and C) latent *money-borrowing* networks. 56

Figure S9 Posterior distributions for the *money-borrowing* model only using network reporters 57

Figure S10 *Social selection* model results – FGMC preference block effects 58

Figure S11 Reciprocity and variation in *marriage-advice* ties 59

Figure S12 *Social selection* model results – focal (out-degree) and target (in-degree) effects.... 60

Figure S13 *Social selection* model – dyadic effects........ 61

Figure S14 *Social selection* model results – gender block effects 62

References 63

Methodology – additional details

Data

The *Household Census* was conducted in 2021, with the aim of visiting each household within the nine zones, censusing occupants aged 15 years or over (referred to as adults from now on). The full names of each adult household occupant and their basic demographic data were collected from one adult member of the household. Each household was assigned a unique four-digit ID, the first number of which signified the zone, and each adult occupant assigned a six-digit individual ID (household ID+01…n); where polygynously married men were reported as the head of multiple households, they were secondarily assigned an individual ID reflecting a concatenation of each of their household IDs. The final data reflects information regarding 5578 individuals, across 1949 households.

The *Norms and Networks Survey* was subsequently conducted in 2021-2022, with the aim of collecting sentiments and perceptions regarding FGMC from all individuals identified in the *Household Census*. The *Norms and Networks Survey* had four variants (1A, 1B, 2A, 2B); in all versions respondents were asked to report their age, educational attainment, gender, the name of the head of their household and their relationship to them, questions about FGMC and in versions 1A and 2A respondents were asked to report their social network connections in four different domains (chatting, respect, money, advice). For each alter (i.e. social tie) named, their age, gender, relationship to the interviewee, and whether they resided in the same zone was also recorded. Sixteen interviewers worked across the zones, with each interviewer responsible for specific households. Each interviewer was given a set of surveys ordered in a repeating pattern of 1A, 1B, 2A, 2B and instructed to administer the surveys in this order to participants as they were available. As such, social network data was collected semi-randomly, within and between households, from approximately half of participants. The respondents interviewed and the alters they named in their networks, where possible, were matched against the *Household Census* to assign individual IDs. Arsi Oromo names take the form of a first name (unique to the individual), a middle name (the first name of the individual's father), and last name (the middle name of the individual's father/first name of the individual's paternal grandfather); names were matched using Levenshtein similarity scores, with candidate IDs further corroborated by cross-checking responses on other characteristics.

Five thousand one hundred and eighty one interviews were conducted and the final sample encompasses 5163 participants (including 50 who were not captured by the *Household Census*), 2545 (49.3 %) of whom reported social network data: two cases were excluded due to unreconcilable recording errors. Sixteen exclusions were made to remove one set of responses where individuals were interviewed twice; where applicable, we retained the variant including social network data and where either two sets of network data or no network data were available we retained the responses from the earliest date. Of 54,632 alters named by respondents in response to six name generator questions, 41,235 (75.5%) were assigned IDs from the *Household Census* and 39,572 (72.4%) were found to have participated in the *Norms and Networks Survey* and are included in the following analyses.

Measures

The following information was delivered to participants of versions 1A and 2A of the *Normas and Networks Survey*:

In our life, each of us, in different ways, interact with other people for advice, support, money and conversation. To get a sense of who you interact with in different parts of your life, we will ask you a

number of questions and ask you to name those people who you interact with in different ways. The same people can be named in answer to the different questions, but they must be 15 years or older.

Participants then received the following name generator questions:

Who do you spend time chatting with? *This could be inside the home or when you're out and about, this could be when you're doing your chores or working, when eating a meal, or walking somewhere. Please give the names of anyone that you spend time chatting with no matter what you're doing. They could be friends, family members, people you work with, or neighbours etc.*

Who would you go to for advice on preparing your daughter for marriage? Or if you do not have a daughter, who would you go to if you did have a daughter. *This could be for advice about a marriage partner, or about dowry or bridewealth arrangements, or anything else to do with preparing a daughter for marriage. Please give the names of the people you would go to for this type of advice. They could be friends, family members, people you work with, or neighbours etc.*

***Who would come to you for advice on preparing their daughter for marriage*?** *This could be advice about a marriage partner, or about dowry or bridewealth arrangements, or anything else to do with preparing a daughter for marriage. Please give the names of the people who would come to you for this type of advice. They could be friends, family members, people you work with, or neighbours etc*

Who do you respect and admire? *These could be people you talk to regularly, or they could be people you do not know well or talk to, but their opinions are important to you. They could be friends, family members, people you work with, or neighbours etc.*

From whom would you feel comfortable asking to borrow 100 birr if you needed it? *Sometimes, for example in an emergency, it might be necessary to borrow money from someone. Please give the names of people you would feel comfortable borrowing 100 birr. They could be friends, family members, people you work with, or neighbours etc.*

Who do you think would be comfortable asking to borrow 100 birr from you if they needed it? *Sometimes, for example in an emergency, someone might need to borrow money from you. Please give the names of people you think would feel comfortable asking to borrow 100 birr from you. They could be friends, family members, people you work with, or neighbours etc.*

Analytical strategy

Social influence – We conducted *post hoc* explorations of models with the inclusion of additional dependencies, allowing for the estimation of other forms of contagion as facilitated by the *BayesALAAM* package: *reciprocal contagion* captures whether a respondent is more likely to be pro-FGMC if they are mutually tied to another respondent who is pro-FGMC; *indirect contagion* captures whether a respondent is more likely to be pro-FGMC if they are indirectly connected to respondents who are pro-FGMC; *closed indirect contagion* captures whether a respondent is more likely to be pro-FGMC if they are both directly and indirectly tied to other respondents who are pro-FGMC, and; *transitive contagion* captures whether a respondent is more likely to be pro-FGMC if they are embedded in triads where the other two members are pro-FGMC. The statistical principle of hierarchy requires that lower-order effects of a given contagion parameter be modelled, thus we made stepwise additions to the original direct contagion models as follows, we added: 1) the reciprocal contagion parameter ($\Sigma_{i<j}Y_iY_jX_{ij}X_{ji}$) and the number of reciprocal ties; 2) the indirect contagion parameter ($\Sigma_iY_i\Sigma_iX_{ij}\,\Sigma_{k\neq i,j}Y_kX_{jk}$), mixed two-path ties as a measure of brokerage, and the number of indirect ties; 3) the closed indirect parameter ($\Sigma_iY_i\Sigma_iX_{ij}\,\Sigma_{k\neq i,j}\,Y_kX_{ik}X_{jk}$) and the number of

indirect ties excluding those that are also direct ties, and; 4) the transitive contagion parameter ($\Sigma_i Y_i \Sigma_i X_{ij} Y_j \Sigma_{k \neq i,j} Y_k X_{ik} X_{jk}$) and the number of embedded transitive triads. Where goodness-of-fit simulations (see below) indicated the model fit was improved by the addition of reciprocal contagion and reciprocal ties in either the reciprocal contagion or indirect contagion models they were retained in subsequent steps, otherwise they were removed. Indirect contagion is a lower-order effect of both closed indirect and transitive contagion, so the parameters added in step 3 were retained for both steps 4 and 5.

Goodness-of-fit simulations were conducted *post hoc* to assess whether the structure of the observed network could be adequately recovered from these models and whether there were dependencies in the data not being captured; in line with standard practise in the ALAAM and exponential random graph model literature, we also include a 'null' logit model with no contagion for comparison. We used the *BayesALAAM* goodness-of-fit function (for details see Koskinen and Daraganova 2022) which returns posterior distributions for a range of dependencies and associated statistics. We present the mean and the 90% highest posterior density interval (HPDI) of the distribution of expected values from a given model for comparison against the observed network statistics. Inferences regarding goodness of fit are based on the distance between the mean and the observed value and the width of the HPDI, with better fit indicated by a smaller distance between the mean and the observation, the observation falling within the HPDI and, where this is the case, a narrower interval. The results of these simulations can be seen in Tables S11-19; the full model results from the best fitting models are presented in Tables S20-22, and the posteriors for the contagion parameters are plotted in Figure S8.

Social selection – The combined stochastic block and social relations models which *STRAND* performs are extremely computationally expensive; for example, the model for zone 8 took just over two weeks to run and produced output files of over 11500MB. As such, we were constrained in the number of chains and iterations we could run. The model performance diagnostics for zone's 2-7 are based 3 chains of 1000 warm-up and 2000 sampling iterations and an adapt delta of 0.98. However, with these specifications the models for largest zones (1 and 8) failed to save, having exceeded the memory limits of the ISEM computing cluster. Hence Zone 1 was run with 1 chain of 1000 warm-up and 2000 sampling iterations and an adapt delta of 0.98; due to warnings in the estimation of Zone 8 under these specifications, it was run with 1500 warm-up and 2500 sampling iterations and an adapt delta of 0.99.

Sample characteristics

While respondents could name up to ten alters in response to each name generator question, not all alters could be identified within the *Household Census* and also took the *Norms and Networks Survey* ensuring we knew their FGMC sentiment too. A comparison of the proportional representation of relationship types among *all nominated* alters with *Norms and Networks Survey-taking* alters indicates that the subsets of the latter (used when modelling *social influence*) and within-zone identified alters who also reported networks (used when modelling *social selection*) are broadly representative of respondents' full reported networks (Table S5). The only relationship categories disproportionately excluded are women's siblings nominated in response to the *advice out* and *advice in* name generators, a likely consequence of patrilocality; the greatest shift being in relation to the proportion of sisters, dropping from 12.4% to 3.6% (*advice in*), and concomitant increase was disproportionately associated with the unrelated friends and neighbours. Proportionally few ties were sent across zone boundaries: *chatting* 2.9% (Figure 1B), *respect* 3.8%, *borrow out* 2.8%, *borrow in* 2.9%, *advice out* 4.0%, and *advice in* 4.3% – those lost did not further alter the representativeness of the *social selection* subsample (Table S5).

Tables

Table S1 Demographic characteristics by kebele-zone

		Kebele zone								
		**1**	**2**	**3**	**4**	**5**	**6**	**7**	**8**	**9**
**n**		1031	400	316	322	633	305	820	1122	214
		Median (IQR)								
**Age (years)**		30 (22)	28 (23.5)	30 (24.25)	30 (29.75)	29 (27)	30 (23)	32 (26)	30 (22)	35 (24)
		Percentage								
**Pro-FGMC**		12.124	9.500	8.861	7.764	7.109	4.262	2.683	2.317	1.412
**Male**		48.788	53.081	47.196	49.684	49.287	49.250	47.205	48.852	51.341
**Highest education**	None	24.442	27.000	27.215	24.534	23.697	27.213	21.707	22.549	27.103
	Some primary	48.109	48.750	49.051	50.311	50.237	45.246	42.805	44.652	46.729
	Completed primary	9.893	10.500	12.025	12.422	10.742	12.459	12.805	11.854	10.280
	Some secondary or beyond	17.556	13.75	11.709	12.733	15.324	15.082	22.683	20.945	15.888
**Community role**	None	92.629	93.250	89.241	93.478	93.049	91.475	89.634	94.474	91.589
	Role	7.371	6.750	9.810	6.522	6.951	8.525	10.366	5.526	8.411
**Religion**	Muslim	99.321	100	96.519	95.963	99.526	91.475	71.707	99.109	99.533
	Orthodox	0.679	0	3.481	4.037	0.474	8.197	28.171	0.713	0.467
	Protestant	0	0	0	0	0	0	0	0.178	0
	Waqeffatta	0	0	0	0	0	0	0.122	0	0
	Other	0	0	0	0	0	0.328	0	0	0
**Perceived level of pro-FGMC preference among men in zone**	0%	61.106	71.250	74.684	77.329	70.616	77.705	71.707	89.394	84.112
	10%	24.927	23.250	16.456	21.429	25.118	16.721	18.537	7.932	11.215
	20%	8.438	3.250	6.013	1.242	2.844	5.246	6.707	1.693	4.206
	30%	3.589	1.500	1.266	0	0.790	0.328	0.854	0.624	0
	40%	0.776	0.500	0.949	0	0.316	0	0.976	0.089	0
	50%	0.970	0	0	0	0.158	0	0.854	0.267	0
	60%	0.097	0.250	0	0	0.158	0	0.244	0	0
	70%	0	0	0	0	0	0	0.122	0	0
	80%	0.097	0	0	0	0	0	0	0	0
	90%	0	0	0	0	0	0	0	0	0
	100%	0	0	0.633	0	0	0	0	0	0.467
**Perceived level of pro-FGMC preference among women in zone**	0%	64.694	74.750	80.063	81.366	72.038	87.541	80.123	93.494	87.850
	10%	24.054	17.750	11.392	17.081	21.011	9.508	15.854	4.456	11.215
	20%	6.111	5.750	4.114	1.242	5.371	2.623	2.683	1.693	0.467
	30%	2.134	1.000	2.215	0.311	0.474	0.328	1.098	0.178	0
	40%	0.970	0.250	0.949	0	0.790	0	0.122	0.089	0
	50%	1.843	0.500	1.266	0	0.316	0	0.122	0.089	0
	60%	0.097	0	0	0	0	0	0	0	0
	70%	0	0	0	0	0	0	0	0	0
	80%	0	0	0	0	0	0	0	0	0
	90%	0.097	0	0	0	0	0	0	0	0
	100%	0	0	0	0	0	0	0	0	0.467
**Anticipated response from zone members if FGMC arranged**	Disapprove	89.913	90.750	99.051	92.547	90.679	84.262	89.878	87.166	86.449
	Think it none of their business	9.408	8.250	0.949	7.143	8.531	15.082	9.634	12.478	13.551
	Approve	0.679	1.000	0	0.311	0.790	0.656	0.488	0.357	0

Table S2 Empirical expectations across kebele-zones

The proportional representation across zones of *empirical expectations* regarding kebele-zone level prevalence of pro-FGMC preference among men (M) and women (W) dependent on the respondent's personal FGMC preference (e.g. 23% of those pro-FGMC think no men in their zone are pro-FGMC, while 66% of those anti-FGMC think no men are pro).

Empirical expectation		Kebele-zone																	
		1		2		3		4		5		6		7		8		9	
		M	W	M	W	M	W	M	W	M	W	M	W	M	W	M	W	M	W
**0%**	**Pro**	0.232	0.261	0.263	0.395	0.107	0.464	0.440	0.640	0.311	0.356	0.385	0.538	0.500	0.591	0.577	0.808	1	1
	**Anti**	0.663	0.700	0.760	0.785	0.809	0.833	0.801	0.828	0.763	0.748	0.795	0.890	0.723	0.807	0.901	0.938	0.839	0.877
**10%**	**Pro**	0.480	0.464	0.579	0.289	0.536	0.107	0.560	0.280	0.533	0.0378	0.385	0.231	0.227	0.227	0.269	0.115	0	0
	**Anti**	0.217	0.210	0.196	0.166	0.128	0.115	0.185	0.162	0.230	0.197	0.158	0.089	0.184	0.157	0.075	0.043	0.114	0.114
**20%**	**Pro**	0.112	0.064	0.105	0.263	0.250	0.036	0	0.040	0.067	0.156	0.154	0.231	0.091	0.045	0.115	0.077	0	0
	**Anti**	0.081	0.061	0.025	0.036	0.042	0.042	0.013	0.010	0.026	0.046	0.048	0.017	0.066	0.026	0.015	0.016	0.043	0.005
**30%**	**Pro**	0.080	0.056	0.026	0.026	0.036	0.179	0	0.040	0.044	0.067	0.077	0	0	0.091	0.038	0	0	0
	**Anti**	0.030	0.017	0.014	0.008	0.010	0.007	0	0	0.005	0	0	0	0.009	0.009	0.005	0.002	0	0
**40%**	**Pro**	0.024	0.040	0.026	0.026	0.036	0.071	0	0	0.044	0.022	0	0	0.045	0.045	0	0	0	0
	**Anti**	0.006	0.006	0.003	0.003	0.007	0.003	0	0	0.002	0.007	0	0	0.009	0	0.001	0.001	0	0
**50%**	**Pro**	0.056	0.096	0	0	0	0.143	0	0	0	0.022	0	0	0.045	0	0	0	0	0
	**Anti**	0.003	0.008	0.003	0.003	0	0	0	0	0.002	0.002	0	0.003	0.008	0.001	0.003	0.001	0	0
**60%**	**Pro**	0.008	0.008	0	0	0	0	0	0	0	0	0	0	0.091	0	0	0	0	0
	**Anti**	0	0	0	0	0	0	0	0	0	0	0	0	0	0	0	0	0	0
**70%**	**Pro**	0.008	0.008	0	0	0	0	0	0	0	0	0	0	0	0	0	0	0	0
	**Anti**	0	0	0	0	0	0	0	0	0	0	0	0	0.001	0	0	0	0	0
**80%**	**Pro**	0	0	0	0	0	0	0	0	0	0	0	0	0	0	0	0	0	0
	**Anti**	0	0	0	0	0	0	0	0	0	0	0	0	0	0	0	0	0	0
**90%**	**Pro**	0	0	0	0	0	0	0	0	0	0	0	0	0	0	0	0	0	0
	**Anti**	0	0	0	0	0	0	0	0	0	0	0	0	0	0	0	0	0	0
**100%**	**Pro**	0	0	0	0	0.036	0	0	0	0	0	0	0	0	0	0	0	0	0
	**Anti**	0	0	0	0	0.003	0	0	0	0	0	0	0	0	0	0	0	0.005	0.005

Table S3 Normative expectations across kebele-zones

The proportional distribution of anticipated reactions by fellow kebele-zone members if a local family arranged FGMC for their daughter, dependent on the respondent's personal FGMC preference.

	Proportional Reaction					
	Disapprove		Think it none of their business		Approve	
**Kebele-zone**	**Pro**	**Anti**	**Pro**	**Anti**	**Pro**	**Anti**
**1**	0.712	0.925	0.264	0.071	0.024	0.004
**2**	0.842	0.914	0.079	0.083	0.003	0.079
**3**	1	0.990	0	0.010	0	0
**4**	0.760	0.939	0.240	0.057	0	0.003
**5**	0.844	0.912	0.111	0.083	0.044	0.005
**6**	0.692	0.849	0.231	0.147	0.077	0.003
**7**	0.818	0.901	0.182	0.094	0	0.005
**8**	0.654	0.877	0.308	0.120	0.038	0.003
**9**	1	0.863	0	0.137	0	0

Table S4 Subsample comparisons

A comparison of the proportional representation of different types of alter named in response to different name generators by respondent gender and subset of the data: *Overall* encompasses the full set of alters named, *Identified* includes all alters matched to an ID in the *Household Census* and who also took the *Norms and Networks Survey* (used in the ALAAMs), and *Identified within zone* includes the further subset of alters who lived in the same zone as the respondent and also reported networks (used in the STRAND models). Notable differences between subsamples bolded.

**Characteristic**		**Chatting**		**Respect**		**Borrow out**		**Borrow in**		**Advice out**		**Advice in**	
Number of ties	Overall	10034		8393		8764		8599		9642		9265	
	Identified	7462		6804		6439		6321		6509		5960	
	Identified within zone	4186		4078		3706		3513		3767		3392	
		**Men**	**Women**	**Men**	**Women**	**Men**	**Women**	**Men**	**Women**	**Men**	**Women**	**Men**	**Women**
Number of ties	Overall	5084	4950	4345	4048	4317	4447	4217	4382	4855	4787	4590	4675
	Identified	3852	3610	3603	3201	3308	3131	3198	3123	3547	2962	3280	2680
	Identified within zone	2243	1943	2227	1851	1969	1737	1849	1664	2141	1626	1939	1453
% father nominations	Overall	5.5	0.9	7.6	2.9	5.9	1.3	4.3	0.9	10.4	4.6	7.6	1.4
	Identified	6.4	0.9	8.0	2.6	6.7	1.2	4.9	0.7	12.4	4.3	9.0	1.5
	Identified within zone	7.2	1.0	8.7	2.9	7.9	1.3	5.6	0.8	13.3	4.5	9.9	1.7
% mother nominations	Overall	4.5	3.3	4.5	3.0	3.7	2.9	3.2	2.7	6.0	5.6	4.1	2.2
	Identified	5.2	3.6	4.7	2.8	4.2	2.9	3.4	2.5	7.0	5.5	4.9	2.1
	Identified within zone	4.9	2.6	4.9	2.8	4.3	2.0	3.4	1.7	5.8	5.7	4.1	1.2
% brother nominations	Overall	26.1	4.3	10.9	2.2	27.6	7.1	28.4	5.9	32.7	**11.7**	34.7	**13.4**
	Identified	24.7	2.7	10.4	1.7	25.2	4.1	27.6	3.7	28.9	**5.6**	31.5	**6.3**
	Identified within zone	26.2	2.4	11.2	1.6	25.2	3.7	28.7	3.2	28.9	**4.6**	31.9	**5.5**
% sister nominations	Overall	0.7	5.0	0.4	1.3	0.9	5.2	1.1	5.7	0.7	6.1	1.0	**12.4**
	Identified	0.3	2.7	0.1	0.4	0.3	2.0	0.6	2.6	0.1	1.9	0.2	**3.6**
	Identified within zone	0.1	1.6	0.1	0.4	0.3	1.4	0.4	2.0	0.1	1.6	0.1	**2.2**
% son nominations	Overall	5.8	5.8	1.5	0.6	4.9	5.9	6.5	5.7	6.2	4.8	7.6	4.9
	Identified	4.9	5.0	1.2	0.5	4.1	5.1	5.5	5.0	5.3	4.9	6.6	5.0
	Identified within zone	4.7	4.6	0.9	0.5	4.0	4.7	4.8	4.9	4.6	4.6	5.4	4.2
% daughter nominations	Overall	0.4	3.8	0.1	0.1	0.2	2.2	0.3	3.5	0.1	0.8	0.3	3.7
	Identified	0.2	2.5	0.1	0	0.1	1.2	0.1	2.5	0.1	0.3	0.2	1.6
	Identified within zone	0.1	1.3	0.1	0	0.2	0.6	0.1	1.3	0	0.2	0.1	1.0
% paternal grandfather	Overall	0.2	0.02	0.4	0.3	0.1	0	0.1	0	0.4	0.2	0.2	0.4
	Identified	0.2	0.03	0.3	0.3	0.1	0	0.03	0	0.3	0.2	0.2	0.1
	Identified within zone	0.1	0.1	0.2	0.4	0.1	0	0.1	0	0.3	0.3	0.1	0.1
% paternal grandmother	Overall	0.1	0.1	0.1	0.4	0.02	0.2	0	0.1	0.1	0.3	0.1	0.02
	Identified	0.1	0.1	0.1	0.4	0.03	0.2	0	0.1	0.1	0.3	0.1	0.04

	Identified within zone	0.04	0	0.04	0.3	0.1	0.1	0	0.	0.2	0.2	0.1	0.1
% paternal uncle	Overall	4.0	0.1	7.7	1.8	4.3	0.5	3.5	0.3	10.5	1.7	8.4	1.2
	Identified	4.2	0.1	7.7	1.8	4.6	0.5	3.8	0.4	10.4	1.2	8.8	1.1
	Identified within zone	4.3	0.1	7.9	2.2	4.4	0.6	3.6	0.3	10.8	1.4	9.2	1.2
% paternal aunt	Overall	0	0.4	0.02	0.3	0.04	0.4	0.1	0.5	0.1	0.3	0.1	0.5
	Identified	0	0.3	0	0.2	0.03	0.4	0.03	0.5	0.1	0.2	0.1	0.4
	Identified within zone	0	0.3	0	0.1	0	0.5	0	0.5	0	0.2	0.1	0.3
% paternal cousin	Overall	2.3	0.6	2.0	0.7	2.7	0.8	2.7	1.0	2.3	0.6	2.4	1.2
	Identified	2.4	0.5	1.9	0.5	2.9	0.6	2.8	0.8	2.4	0.4	2.4	1.1
	Identified within zone	2.8	0.5	1.7	0.3	2.9	0.3	2.8	0.5	2.6	0.2	2.6	1.2
% maternal grandfather	Overall	0.1	0.02	0.4	0.2	0.1	0.02	0.1	0.1	0.2	0.04	0.4	0
	Identified	0.1	0	0.5	0.2	0.2	0	0.2	0.1	0.3	0.1	0.4	0
	Identified within zone	0.2	0.1	0.4	0	0.1	0	0.1	0	0.2	0.1	0.4	0
% maternal grandmother	Overall	0.03	0.02	0.1	0.2	0.1	0.1	0.1	0.1	0.04	0.2	0	0.1
	Identified	0.1	0	0.1	0.2	0.1	0.1	0.1	0.03	0.1	0.2	0	0.1
	Identified within zone	0.04	0	0.04	0.1	0.1	0.1	0.1	0	0.1	0.1	0	0.1
% maternal uncle	Overall	0.5	0.2	0.9	0.6	0.8	0.4	0.6	0.1	0.8	1.0	0.7	0.7
	Identified	0.3	0.2	0.8	0.5	0.6	0.1	0.4	0.1	0.5	1.2	0.4	0.5
	Identified within zone	0.3	0.1	0.6	0.6	0.6	0.1	0.4	0.1	0.4	0.4	0.4	0.3
% maternal aunt	Overall	0	0.2	0	0.2	0.02	0.2	0.02	0.3	0.1	0.5	0.02	0.6
	Identified	0	0.2	0	0.1	0	0.2	0	0.3	0.03	0.2	0	0.3
	Identified within zone	0	0.2	0	0.1	0	0.2	0	0.2	0	0.1	0	0.1
% maternal cousin	Overall	0.3	0.4	0.3	0.3	0.4	0.4	0.4	0.4	0.3	0.3	0.3	0.5
	Identified	0.3	0.4	0.2	0.2	0.4	0.3	0.3	0.3	0.3	0.2	0.3	0.3
	Identified within zone	0.2	0.4	0.2	0.3	0.3	0.2	0.2	0.4	0.1	0.1	0.2	0.3
% husband	Overall	NA	7.9	NA	4.2	NA	1.5	NA	1.2	NA	6.5	NA	1.9
	Identified	NA	9.5	NA	4.8	NA	1.8	NA	1.3	NA	9.3	NA	2.9
	Identified within zone	NA	10.7	NA	5.4	NA	2.2	NA	1.4	NA	10.6	NA	2.7
% wife	Overall	5.3	NA	2.0	NA	0.7	NA	0.6	NA	4.0	NA	2.0	NA
	Identified	6.1	NA	2.2	NA	0.8	NA	0.8	NA	4.7	NA	2.4	NA
	Identified within zone	6.2	NA	2.2	NA	0.8	NA	0.9	NA	4.4	NA	2.1	NA
% co-wife	Overall	NA	1.4	NA	0.5	NA	1.1	NA	1.3	NA	0.8	NA	1.0
	Identified	NA	1.6	NA	0.6	NA	1.3	NA	1.5	NA	1.0	NA	1.6
	Identified within zone	NA	1.2	NA	0.6	NA	1.2	NA	1.7	NA	1.0	NA	1.5
% daughter-in-law	Overall	0	2.7	0.02	0.4	0	2.2	0.02	2.6	0	0.7	0	1.3
	Identified	0	3.2	0.03	0.4	0	2.8	0.03	3.3	0	0.9	0	2.1
	Identified within zone	0	2.9	0	0.3	0	2.6	0	2.8	0	0.8	0	1.8
% son-in-law	Overall	0.1	0.04	0.04	0.1	0.1	0.1	0.1	0.1	0.02	0.1	0	0.1
	Identified	0.1	0.03	0.1	0.1	0.1	0.1	0.1	0.03	0.03	0.1	0	0.1

	Identified within zone	0.1	0.1	0	0.1	0.1	0.1	0.1	0.1	0	0.1	0	0.3
% sister's husband	Overall	0.5	0.1	0.9	0.2	0.6	0.2	0.4	0.2	2.2	0.2	2.3	0.1
	Identified	0.4	0.1	1.0	0.1	0.7	0.2	0.3	0.1	1.7	0.1	1.6	0.1
	Identified within zone	0.5	0.2	1.2	0.1	0.7	0.2	0.5	0.1	2.2	0	2.2	0.1
% brother's wife	Overall	0.1	0.8	0.02	0.1	0.2	0.8	0.2	0.9	0.1	0.4	0.1	0.6
	Identified	0.1	0.9	0.03	0.1	0.2	1.0	0.3	0.9	0.1	0.4	0.03	0.9
	Identified within zone	0.1	0.9	0.04	0.1	0.2	1.0	0.2	1.1	0.1	0.6	0.1	1.0
% spouse's father	Overall	0.1	0.6	0.2	3.6	0.2	1.0	1.1	0.9	0.2	2.8	0.2	0.9
	Identified	0.1	0.8	0.2	3.9	0.2	1.1	0.2	1.2	0.1	3.7	0.2	1.4
	Identified within zone	0.2	1.0	0.3	4.5	0.2	1.2	0.3	1.3	0.2	4.6	0.3	1.8
% spouse's mother	Overall	0.04	4.9	0.02	4.4	0.1	4.2	0.1	4.7	0.1	4.0	0.1	2.5
	Identified	0.1	6.0	0.03	4.8	0.1	5.2	0.1	6.1	0.1	5.7	0.03	3.8
	Identified within zone	0.04	4.6	0.04	2.9	0.1	4.1	0.1	4.9	0.1	4.4	0.1	3.2
% spouse's brother	Overall	0.4	2.6	0.3	7.2	0.6	7.9	0.5	5.7	1.0	16.1	0.7	9.8
	Identified	0.5	2.4	0.2	7.3	0.7	7.9	0.5	6.0	0.8	16.2	0.7	11.3
	Identified within zone	0.6	2.8	0.3	8.0	0.6	8.4	0.5	6.4	0.8	17.2	0.7	13.1
% spouse's sister	Overall	0.02	2.3	0	0.8	0.04	2.0	0	2.3	0	1.9	0.02	2.8
	Identified	0.03	2.3	0	0.5	0	1.8	0	2.3	0	1.1	0.03	2.1
	Identified within zone	0.03	1.9	0	0.5	0	1.6	0	1.7	0	1.0	0.1	1.5
% niece/nephew	Overall	3.3	0.3	1.9	0.2	2.9	0.5	3.2	0.4	2.1	0.3	2.5	0.3
	Identified	3.4	0.3	1.9	0.2	3.2	0.5	3.4	0.4	2.3	0.3	2.6	0.3
	Identified within zone	3.3	0.3	1.5	0.2	2.7	0.5	3.2	0.5	2.3	0.4	2.3	0.1
% grandchild	Overall	0.1	0.1	0.02	0	0.1	0.04	0.1	0.02	0.1	0	0.2	0.1
	Identified	0.1	0.03	0	0	0.1	0.03	0.1	0.03	0.1	0	0.1	0.1
	Identified within zone	0	0	0	0	0	0	0	0.1	0	0	0.1	0
% other relative	Overall	9.3	6.5	12.8	12.4	9.7	8.7	9.3	7.8	3.4	7.9	4.0	7.5
	Identified	8.9	7.0	12.9	13.0	9.3	9.8	8.9	8.6	3.8	9.4	4.5	9.8
	Identified within zone	8.7	8.2	14.9	12.9	9.6	11.1	9.0	9.3	4.4	10.1	5.0	10.4
% friend/neighbour (non-relative)	Overall	29.8	**44.3**	37.0	49.0	31.2	**42.1**	32.9	44.3	15.1	**19.6**	19.7	**28.0**
	Identified	30.3	**46.7**	38.5	50.1	33.4	**47.6**	34.4	48.7	17.6	**25.5**	22.3	**39.3**
	Identified within zone	28.4	**49.5**	37.0	51.9	32.5	**49.9**	33.6	49.1	17.7	**26.6**	22.3	**42.9**
% other non-relative	Overall	0.6	0.3	7.8	1.8	1.7	0.2	1.3	0.2	0.6	0.3	0.4	0.2
	Identified	0.6	0.3	7.1	1.5	2.0	0.3	1.2	0.3	0.6	0.3	0.4	0.3
	Identified within zone	0.7	0.3	6.3	1.1	2.0	0	1.2	0.2	0.6	0.2	0.4	0.2

Table S5 Measures of network centrality by relationship type dependent on FGMC-preference

Characteristic	Chatting		Respect		Money-borrowing		Marriage-advice	
	Pro-FGMC	Anti-FGMC	Pro-FGMC	Anti-FGMC	Pro-FGMC	Anti-FGMC	Pro-FGMC	Anti-FGMC
**Mean/median in-degree**	1.489/1	1.442/1	1.695/0	1.292/0	1.772/1	1.686/1	1.969/1	1.732/1
**Range in-degree**	0-9	0-11	0-28	0-176	0-8	0-15	0-9	0-19
**Mean/median out-degree**	1.594/0	1.435/0	1.394/0	1.313/0	1.655/1	1.694/1	2.037/1	1.728/1
**Range out-degree**	0-7	0-9	0-6	0-8	0-10	0-69	0-15	0-20
**Mean/median vertex betweenness**	335.759/0	476.305/0	179.279/0	83.720/0	19306.280/1	24145.080/3	19564.67/1.5	18860.34/0
**Range vertex betweenness**	0-27753.04	0-38219.24	0-10021.83	0-16957.06	0-609094.3	0-1543643	0-443339.5	0-735463.8
**Mean/median harmonic centrality**	8.236/0	7.501/0	4.487/0	5.252/0	140.820/144.373	138.774/161.632	142.923/197.202	125.929/9.335
**Range harmonic centrality**	0-81.479	0-94.497	0-25.6	0-28.238	0-463.030	0-518.713	0-385.369	0-404.175

Table S6 Kebele-zone-level network characteristics and pro-FGMC preference prevalence

	1	2	3	4	5	6	7	8	9
n	509	200	159	158	301	154	411	551	102
Proportion pro-FGMC	0.114	0.11	0.107	0.07	0.083	0.045	0.022	0.024	0.029
Chatting									
Density	0.003	0.008	0.011	0.010	0.005	0.011	0.004	0.003	0.014
Reciprocity	0.264	0.283	0.343	0.339	0.312	0.360	0.358	0.341	0.392
Transitivity	0.186	0.277	0.357	0.232	0.285	0.239	0.227	0.250	0.237
Mean/ median total degree	3.191/ 3	3.18/ 2	3.371/ 3	3.215/ 3	3.150/ 3	3.390/ 3	3.557/ 3	3.339/ 3	2.902/ 3
Mean/ median vertex betweenness	550.982 /2	26.305/ 1	38.038/ 0	23.108/ 2.417	16.581/ 0	32.312/ 2.25	993.835 /12	101.054 /5	21.618/ 0
Mean/ median harmonic centrality	11.590/ 13.596	3.913/ 3.258	4.163/ 4	3.671/ 3.5	3.372/ 3	4.647/ 4.318	12.186/ 11.530	5.347/ 4	3.351/2
Respect									
Density	0.004	0.010	0.010	0.009	0.006	0.009	0.004	0.002	0.013
Reciprocity	0.031	0.057	0.065	0.063	0.059	0.058	0.042	0.052	0.058
Transitivity	0.062	0.115	0.141	0.148	0.092	0.133	0.096	0.057	0.131
Mean/ median total degree	3.780/ 2	3.84/2	3.119/ 2	2.835/ 2	3.801/ 3	2.675/ 2	2.988/ 2	2.646/ 2	2.725/ 2
Mean/ median vertex betweenness	45.344/ 0	18.425/ 0	26.270/ 0	15.766/ 0	50.897/ 0	6.032/ 0	179.287 /0	67.831/ 0	5.284/ 0
Mean/ median harmonic centrality	6.123/ 6.417	4.476/ 4.46	4.096/ 3.667	2.677/ 2.773	5.643/ 6.083	2.463/ 2.208	6.6/ 7.836	3.978/ 3.117	2.617/ 2.333
Money-borrowing									
Density	0.004	0.009	0.012	0.013	0.006	0.011	0.005	0.003	0.016
Reciprocity	0.465	0.550	0.583	0.446	0.453	0.428	0.465	0.458	0.618
Transitivity	0.114	0.181	0.223	0.174	0.146	0.138	0.126	0.156	0.169
Mean/ median total degree	4.511/ 3	3.78/ 3	3.711/ 3	4.139/ 3.5	3.429/ 3	3.338/ 3	4.102/ 3	3.568/ 3	3.235/ 3
Mean/ median vertex betweenness	1796.157/ 428	173.215 / 16.833	336.962 / 11	372.196 / 106	510.193 / 4	70.149/ 4	1117.616/ 161.704	757.880 / 18	72.559/ 0
Mean/ median harmonic centrality	51.530/ 62.551	10.184/ 8.418	10.851/ 12.218	17.621/ 21.271	13.691/ 5.083	6.430/ 4.271	32.240/ 39.136	14.317/ 3.5	6.272/ 5.948
Marriage-advice									
Density	0.004	0.010	0.013	0.013	0.006	0.014	0.005	0.003	0.019
Reciprocity	0.520	0.620	0.641	0.491	0.591	0.445	0.471	0.371	0.510
Transitivity	0.236	0.253	0.255	0.232	0.324	0.219	0.249	0.253	0.252
Mean/ median total degree	4.326/ 3	3.97/ 2.5	4.201/ 3	4.127/ 3	3.641/ 3	4.143/ 3	4.462/ 4	3.837/ 3	3.843/ 3
Mean/ median vertex betweenness	1674.024/ 102.107	226.245 /0	353.094 /0	175.652 /9.25	45.306/ 0	317.065 /30.817	1405.409/126	423.681 /2	49.755/ 0.683

Mean/ median harmonic centrality	36.935/ 48.511	11.539/ 4.833	13.054/ 14.488	11.303/ 7.757	6.018/ 3	12.850/ 16.235	27.911/ 36.337	11.985/ 3.5	6.578/ 4.117

Table S7 Latent *money-borrowing* network characteristics

Latent *money-borrowing* network characteristics dependent on whether the network was estimated using the sample of all respondents to the *Norms and Networks Survey* (n = 5163) or the subsample of only those who also reported their network ties (n = 2545).

**Characteristic**	**All respondents**		**Network reporters only**	
**Ties**	8733		5046	
**Density**	0.0003		0.001	
**Reciprocity**	0.422		0.471	
**Transitivity**	0.121		0.136	
**Isolates**	1201		144	
	**Pro-FGMC**	**Anti-FGMC**	**Pro-FGMC**	**Anti-FGMC**
**Mean/median in-degree**	1.772/1	1.686/1	2.121/2	1.973/2
**Range in-degree**	0-8	0-15	0-8	0-15
**Mean/median out-degree**	1.655/1	1.694/1	2.097/2	1.975/1
**Range out-degree**	0-10	0-69	0-9	0-39
**Mean/median vertex betweenness**	19306.280/ 1	24145.080/ 3	19275.78/ 1395.5	14155.05/ 224
**Range vertex betweenness**	0-609094.3	0-1543643	0-416459.2	0-943448.2
**Mean/median harmonic centrality**	140.820/ 144.373	138.774/ 161.632	77.507/ 78.927	67.808/ 67.347
**Range harmonic centrality**	0-463.030	0-518.713	0-224.103	0-257.520

Table S8 Full results from the ALAAM models exploring contagion, i.e. *social influence*, in the estimated *chatting* networks

Parameter	Mean	sd	Credible interval	
			**0.025**	**0.975**
All ties				
**Intercept**	-1.788	0.298	-2.361	-1.157
**Contagion**	**0.501**	**0.144**	**0.207**	**0.77**
**Gender**	-0.031	0.207	-0.441	0.375
**Age**	0.016	0.006	0.004	0.028
**Some primary vs none**	-0.416	0.246	-0.886	0.082
**Completed primary vs none**	-0.208	0.382	-0.955	0.538
**Some secondary vs none**	-0.706	0.394	-1.519	0.069
**Zone 2 vs 1**	-0.231	0.306	-0.856	0.324
**Zone 3 vs 1**	-0.314	0.361	-1.054	0.396
**Zone 4 vs 1**	-0.437	0.343	-1.135	0.237
**Zone 5 vs 1**	-0.507	0.3	-1.106	0.067
**Zone 6 vs 1**	-1.1	0.56	-2.251	-0.116
**Zone 7 vs 1**	-1.489	0.42	-2.388	-0.716
**Zone 8 vs 1**	-1.601	0.376	-2.397	-0.897
**Zone 9 vs 1**	-2.666	1.471	-6.984	-0.732
**Out-degree**	-0.03	0.062	-0.153	0.094
**In-degree**	-0.111	0.068	-0.251	0.02
Kin-only ties				
**Intercept**	-1.739	0.288	-2.316	-1.197
**Contagion**	**0.445**	**0.194**	**0.055**	**0.817**
**Gender**	-0.039	0.205	-0.449	0.352
**Age**	0.016	0.006	0.003	0.028
**Some primary vs none**	-0.393	0.26	-0.922	0.095
**Completed primary vs none**	-0.119	0.345	-0.824	0.522
**Some secondary vs none**	-0.716	0.381	-1.491	-0.004
**Zone 2 vs 1**	-0.225	0.302	-0.832	0.35
**Zone 3 vs 1**	-0.419	0.365	-1.178	0.285
**Zone 4 vs 1**	-0.534	0.384	-1.311	0.187
**Zone 5 vs 1**	-0.575	0.318	-1.262	0.025
**Zone 6 vs 1**	-1.19	0.512	-2.255	-0.258
**Zone 7 vs 1**	-1.627	0.394	-2.421	-0.9
**Zone 8 vs 1**	-1.706	0.382	-2.535	-1.016
**Zone 9 vs 1**	-2.903	1.529	-7.352	-1
**Out-degree**	-0.055	0.079	-0.211	0.1
**In-degree**	-0.101	0.088	-0.279	0.066
Non-kin-only ties				
**Intercept**	-1.809	0.261	-2.335	-1.289
**Contagion**	**0.650**	**0.248**	**0.137**	**1.133**
**Gender**	-0.017	0.198	-0.399	0.374
**Age**	0.016	0.006	0.003	0.027
**Some primary vs none**	-0.410	0.230	-0.878	0.024

**Completed primary vs none**	-0.219	0.369	-0.955	0.495
**Some secondary vs none**	-0.692	0.367	-1.471	-0.029
**Zone 2 vs 1**	-0.283	0.304	-0.918	0.287
**Zone 3 vs 1**	-0.324	0.377	-1.125	0.353
**Zone 4 vs 1**	-0.479	0.368	-1.276	0.208
**Zone 5 vs 1**	-0.580	0.312	-1.204	0.005
**Zone 6 vs 1**	-1.069	0.467	-2.091	-0.263
**Zone 7 vs 1**	-1.573	0.384	-2.400	-0.868
**Zone 8 vs 1**	-1.731	0.342	-2.436	-1.105
**Zone 9 vs 1**	-2.454	0.922	-4.583	-0.880
**Out-degree**	0.004	0.090	-0.179	0.180
**In-degree**	-0.156	0.110	-0.375	0.052

Table S9 Full results from the ALAAM models exploring contagion, i.e. *social influence*, in the estimated *respect* networks

**Parameter**	**Mean**	**sd**	**Credible interval**	
			**0.025**	**0.975**
All ties				
**Intercept**	-2.338	0.42	-3.198	-1.545
**Contagion**	**0.306**	**0.154**	**0.002**	**0.601**
**Gender**	-0.046	0.205	-0.449	0.367
**Age**	0.017	0.007	0.004	0.03
**Some primary vs none**	-0.374	0.228	-0.83	0.061
**Completed primary vs none**	-0.164	0.387	-1.013	0.548
**Some secondary vs none**	-0.719	0.353	-1.432	0.001
**Zone 2 vs 1**	-0.315	0.331	-0.984	0.295
**Zone 3 vs 1**	-0.413	0.356	-1.162	0.248
**Zone 4 vs 1**	-0.543	0.38	-1.333	0.157
**Zone 5 vs 1**	-0.528	0.281	-1.088	0.038
**Zone 6 vs 1**	-1.158	0.481	-2.148	-0.278
**Zone 7 vs 1**	-1.591	0.393	-2.366	-0.876
**Zone 8 vs 1**	-1.726	0.381	-2.521	-1.003
**Zone 9 vs 1**	-3.087	1.388	-6.265	-1.013
**Out-degree**	-0.041	0.067	-0.179	0.083
**In-degree**	-0.052	0.033	-0.123	0.005
Kin-only ties				
**Intercept**	-2.276	0.416	-3.096	-1.485
**Contagion**	**0.211**	**0.239**	**-0.279**	**0.655**
**Gender**	-0.073	0.199	-0.47	0.296
**Age**	0.017	0.006	0.004	0.029
**Some primary vs none**	-0.407	0.253	-0.903	0.1
**Completed primary vs none**	-0.201	0.358	-0.872	0.513
**Some secondary vs none**	-0.749	0.402	-1.529	0.01
**Zone 2 vs 1**	-0.255	0.306	-0.919	0.283
**Zone 3 vs 1**	-0.452	0.385	-1.255	0.229
**Zone 4 vs 1**	-0.607	0.385	-1.361	0.108
**Zone 5 vs 1**	-0.563	0.285	-1.124	-0.012
**Zone 6 vs 1**	-1.238	0.531	-2.399	-0.334
**Zone 7 vs 1**	-1.586	0.369	-2.301	-0.898
**Zone 8 vs 1**	-1.799	0.364	-2.5	-1.096
**Zone 9 vs 1**	-2.971	1.27	-6.044	-1.043
**Out-degree**	-0.037	0.087	-0.21	0.13
**In-degree**	-0.053	0.058	-0.184	0.043
Non-kin-only ties				
**Intercept**	-2.343	0.41	-3.164	-1.558
**Contagion**	**0.446**	**0.258**	**-0.059**	**0.953**
**Gender**	-0.065	0.211	-0.495	0.357
**Age**	0.017	0.006	0.006	0.029
**Some primary vs none**	-0.395	0.256	-0.866	0.119

**Completed primary vs none**	-0.154	0.388	-0.94	0.594
**Some secondary vs none**	-0.7	0.382	-1.47	0.044
**Zone 2 vs 1**	-0.309	0.329	-1.011	0.285
**Zone 3 vs 1**	-0.425	0.387	-1.28	0.272
**Zone 4 vs 1**	-0.549	0.35	-1.239	0.096
**Zone 5 vs 1**	-0.583	0.284	-1.18	-0.052
**Zone 6 vs 1**	-1.196	0.501	-2.323	-0.328
**Zone 7 vs 1**	-1.627	0.404	-2.496	-0.896
**Zone 8 vs 1**	-1.738	0.399	-2.577	-1
**Zone 9 vs 1**	-2.669	1.104	-5.15	-0.912
**Out-degree**	-0.054	0.094	-0.251	0.133
**In-degree**	-0.091	0.052	-0.201	-0.004

Table S10 Full results from the ALAAM models exploring contagion, i.e. *social influence*, in the latent *money-borrowing* networks

Estimated either using the full sample of identified alters who were respondents in the *Norms and Networks Survey* or only those alters who also reported their networks.

Parameter	All Norms and Networks Survey respondents				Norms and Networks Survey network reporters only			
			Credible interval				Credible interval	
	**Mean**	**sd**	**0.025**	**0.975**	**Mean**	**sd**	**0.025**	**0.975**
All ties								
**Intercept**	-1.715	0.294	-2.327	-1.143	-1.824	0.365	-2.604	-1.175
**Contagion**	**0.272**	**0.143**	**-0.034**	**0.519**	**0.421**	**0.121**	**0.182**	**0.655**
**Gender**	-0.054	0.205	-0.456	0.347	0.070	0.247	-0.382	0.568
**Age**	0.016	0.006	0.005	0.028	0.019	0.007	0.006	0.033
**Some primary vs none**	-0.378	0.240	-0.832	0.096	-0.572	0.274	-1.129	-0.048
**Completed primary vs none**	-0.141	0.367	-0.885	0.567	-0.036	0.390	-0.823	0.691
**Some secondary vs none**	-0.686	0.384	-1.502	0.025	-0.539	0.416	-1.321	0.317
**Zone 2 vs 1**	-0.251	0.320	-0.912	0.334	-0.062	0.352	-0.800	0.568
**Zone 3 vs 1**	-0.371	0.349	-1.106	0.292	-0.122	0.367	-0.913	0.551
**Zone 4 vs 1**	-0.503	0.398	-1.326	0.191	-0.548	0.440	-1.438	0.290
**Zone 5 vs 1**	-0.556	0.276	-1.131	-0.040	-0.376	0.298	-1.002	0.162
**Zone 6 vs 1**	-1.132	0.518	-2.231	-0.214	-0.877	0.495	-1.908	-0.023
**Zone 7 vs 1**	-1.603	0.421	-2.506	-0.873	-1.748	0.462	-2.694	-0.912
**Zone 8 vs 1**	-1.694	0.380	-2.482	-0.978	-1.588	0.400	-2.411	-0.841
**Zone 9 vs 1**	-2.733	1.215	-5.605	-0.945	-1.672	0.864	-3.629	-0.328
**Out-degree**	-0.119	0.076	-0.284	0.021	-0.086	0.075	-0.245	0.054
**In-degree**	0.008	0.085	-0.167	0.167	-0.058	0.089	-0.227	0.117
Kin-only ties								
**Intercept**	-1.699	0.286	-2.283	-1.151	-1.915	0.345	-2.643	-1.290
**Contagion**	**0.356**	**0.181**	**0.004**	**0.703**	**0.495**	**0.179**	**0.131**	**0.831**
**Gender**	-0.089	0.214	-0.512	0.331	0.041	0.226	-0.406	0.482
**Age**	0.017	0.006	0.005	0.028	0.021	0.007	0.007	0.035
**Some primary vs none**	-0.407	0.234	-0.879	0.051	-0.480	0.246	-0.949	-0.003
**Completed primary vs none**	-0.152	0.372	-0.959	0.507	0.011	0.416	-0.807	0.826
**Some secondary vs none**	-0.730	0.381	-1.484	-0.015	-0.590	0.428	-1.443	0.213
**Zone 2 vs 1**	-0.281	0.307	-0.892	0.355	-0.050	0.344	-0.722	0.622
**Zone 3 vs 1**	-0.448	0.354	-1.150	0.241	-0.219	0.365	-0.979	0.473
**Zone 4 vs 1**	-0.606	0.388	-1.388	0.120	-0.606	0.442	-1.496	0.234
**Zone 5 vs 1**	-0.600	0.299	-1.175	-0.043	-0.386	0.318	-1.021	0.224
**Zone 6 vs 1**	-1.203	0.499	-2.247	-0.279	-0.998	0.508	-2.097	-0.074
**Zone 7 vs 1**	-1.651	0.380	-2.444	-0.932	-1.784	0.488	-2.835	-0.865
**Zone 8 vs 1**	-1.754	0.402	-2.621	-1.027	-1.593	0.401	-2.394	-0.858
**Zone 9 vs 1**	-2.832	1.214	-5.561	-0.881	-1.715	0.830	-3.619	-0.341
**Out-degree**	-0.143	0.076	-0.296	0.000	-0.147	0.077	-0.302	-0.005
**In-degree**	0.021	0.066	-0.116	0.150	0.027	0.091	-0.158	0.204

Non-kin-only ties								
**Intercept**	-1.713	0.290	-2.365	-1.186	-1.854	0.291	-2.447	-1.282
**Contagion**	**0.509**	**0.210**	**0.052**	**0.888**	**0.438**	**0.218**	**-0.008**	**0.849**
**Gender**	-0.049	0.199	-0.436	0.347	-0.008	0.231	-0.470	0.439
**Age**	0.017	0.006	0.006	0.029	0.020	0.007	0.007	0.033
**Some primary vs none**	-0.395	0.242	-0.855	0.086	-0.576	0.235	-1.053	-0.137
**Completed primary vs none**	-0.131	0.376	-0.838	0.629	-0.002	0.385	-0.793	0.718
**Some secondary vs none**	-0.699	0.363	-1.431	-0.031	-0.520	0.393	-1.312	0.240
**Zone 2 vs 1**	-0.297	0.299	-0.885	0.274	-0.053	0.325	-0.678	0.572
**Zone 3 vs 1**	-0.402	0.392	-1.209	0.304	-0.093	0.383	-0.921	0.672
**Zone 4 vs 1**	-0.493	0.401	-1.321	0.225	-0.613	0.442	-1.546	0.213
**Zone 5 vs 1**	-0.565	0.297	-1.166	-0.005	-0.388	0.309	-1.052	0.210
**Zone 6 vs 1**	-1.253	0.565	-2.500	-0.288	-1.102	0.578	-2.367	-0.097
**Zone 7 vs 1**	-1.637	0.424	-2.476	-0.814	-1.765	0.441	-2.678	-0.967
**Zone 8 vs 1**	-1.709	0.365	-2.466	-1.022	-1.675	0.386	-2.514	-0.949
**Zone 9 vs 1**	-2.743	1.166	-5.565	-0.915	-1.720	0.840	-3.660	-0.368
**Out-degree**	-0.176	0.094	-0.373	-0.007	-0.207	0.095	-0.423	-0.040
**In-degree**	-0.023	0.077	-0.172	0.120	0.102	0.099	-0.103	0.291

Table S11 Goodness-of-fit results for models exploring *social influence* in the all alters *chatting* networks (the best fitting model is indicated in bold)

Parameter	Observed	Null			Direct			Reciprocal		
		Mean	HPDI		Mean	HPDI		Mean	HPDI	
			LI	UI		LI	UI		LI	UI
**Intercept**	325	322.932	288	355	**354.336**	**303**	**398**	330.232	296	362
**Direct contagion**	82	49.844	34	68	**78.02**	**42**	**107**	63.464	37	93
**Reciprocal contagion**	8	4.778	2	8	**8.664**	**3**	**14**	5.592	2	10
**Indirect contagion**	117	84.076	54	112	**107.502**	**51**	**163**	95.274	58	137
**Closed indirect contagion**	35	18.530	8	28	**30.62**	**11**	**47**	22.52	9	38
**Transitive contagion**	15	2.556	0	6	**9.186**	**0**	**19**	5.118	0	13
**Out-degree**	518	531.688	446	610	**542.122**	**454**	**662**	550.95	472	644
**In-degree**	484	495.376	420	577	**509.146**	**376**	**606**	484.386	415	552
**Reciprocal ties**	85	92.712	72	113	**91.596**	**62**	**113**	80.284	58	104
**In-two star**	572	595.240	440	741	**577.258**	**345**	**786**	571.276	404	730
**Out-two star**	684	662.872	550	776	**663.942**	**536**	**799**	686.82	576	823
**Mixed two-path**	838	926.370	738	1134	**883.578**	**642**	**1143**	894.642	716	1067
**In-three star**	592	658.842	388	944	**613.438**	**250**	**967**	634.928	308	952
**Out-three star**	477	417.394	323	504	**411.57**	**322**	**537**	431.986	331	518
**Transitive triads**	184	189.258	146	222	**186.85**	**142**	**248**	180.404	133	220
**Cyclic**	67	71.948	49	93	**69.832**	**43**	**96**	60.476	37	79
**Indirect ties**	945	907.140	732	1038	**927.034**	**698**	**1154**	935.042	788	1086
**Exclusively indirect ties**	714	671.454	556	784	**693.408**	**539**	**873**	708.824	598	831
**Parameter**	**Observed**	**Indirect**			**Closed indirect**			**Transitive**		
		**Mean**	**HPDI**		**Mean**	**HPDI**		**Mean**	**HPDI**	
			**LI**	**UI**		**LI**	**UI**		**LI**	**UI**
**Intercept**	325	361.462	307	446	324.574	291	354	336.196	294	377
**Direct contagion**	82	147.016	56	333	50.826	28	70	131.798	45	223
**Reciprocal contagion**	8	19.902	5	52	4.274	0	7	18.93	2	32
**Indirect contagion**	117	350.238	75	882	93.514	52	146	322.404	73	604
**Closed indirect contagion**	35	91.682	12	232	16.98	7	29	85.842	13	143
**Transitive contagion**	15	64.8	0	196	2.632	0	7	59.822	0	114
**Out-degree**	518	602.568	529	918	486.836	412	552	574.856	443	703
**In-degree**	484	588.118	429	844	493.274	417	573	569.458	438	673
**Reciprocal ties**	85	110.83	80	187	80.696	62	102	109.626	77	137
**In-two star**	572	802.448	563	1472	579.972	398	716	825.356	498	1100
**Out-two star**	684	766.102	655	1219	595.924	501	690	740.804	545	949
**Mixed two-path**	838	1119.86	962	2050	773.15	578	942	1181.386	791	1604
**In-three star**	592	1035.992	629	2249	636.464	346	918	1145.13	509	1759
**Out-three star**	477	494.642	468	883	367.568	285	445	491.13	323	656
**Transitive triads**	184	242.006	171	415	157.466	121	191	227.65	154	284
**Cyclic**	67	112.886	61	225	57.694	31	78	114.81	58	158
**Indirect ties**	945	1177.392	927	1897	893.33	754	1044	1110.102	794	1405
**Exclusively indirect ties**	714	863.39	701	1373	688.406	563	797	815.342	602	1059

Table S12 Goodness-of-fit results for models exploring *social influence* in the kin only *chatting* networks (the best fitting model is indicated in bold)

Parameter	Observed	Null			Direct			Reciprocal		
		Mean	HPDI		Mean	HPDI		Mean	HPDI	
			LI	UI		LI	UI		LI	UI
**Intercept**	325	308.144	277	336	337.298	302	370	318.004	286	344
**Direct contagion**	42	22.960	13	35	60.188	35	83	22.062	12	31
**Reciprocal contagion**	6	2.244	0	4	8.45	3	14	2.196	0	5
**Indirect contagion**	42	29.102	11	42	73.972	30	114	25.658	13	38
**Closed indirect contagion**	15	8.324	2	15	27.488	9	47	6.934	1	12
**Transitive contagion**	9	1.040	0	3	11.748	0	25	0.698	0	2
**Out-degree**	291	287.188	239	325	331.592	273	380	269.504	224	305
**In-degree**	289	281.024	230	326	332.676	250	389	290.268	243	337
**Reciprocal ties**	52	54.092	39	66	66.074	50	85	52.782	39	64
**In-two star**	228	231.438	171	298	318.354	176	441	238.566	150	316
**Out-two star**	286	267.204	218	318	314.276	247	379	238.314	183	288
**Mixed two-path**	306	347.542	275	441	452.442	321	595	318.226	229	410
**In-three star**	127	190.868	86	303	314.152	77	487	199.064	68	328
**Out-three star**	175	135.596	101	176	165.16	112	209	117.376	75	150
**Transitive triads**	87	95.628	67	119	114.316	80	151	83.104	60	104
**Cyclic**	32	38.512	22	53	55.16	31	82	33.588	19	48
**Indirect ties**	346	346.510	272	406	430.914	325	519	313.306	257	378
**Exclusively indirect ties**	244	232.080	177	270	290.842	233	357	213.56	174	262
**Parameter**	**Observed**	**Indirect**			**Closed indirect**			**Transitive**		
		**Mean**	**HPDI**		**Mean**	**HPDI**		**Mean**	**HPDI**	
			**LI**	**UI**		**LI**	**UI**		**LI**	**UI**
**Intercept**	325	**335.9**	**291**	**376**	375.228	338	406	356.068	303	412
**Direct contagion**	42	**38.63**	**16**	**59**	135.088	28	206	86.676	26	157
**Reciprocal contagion**	6	**3.958**	**0**	**7**	24.368	4	47	16.22	0	36
**Indirect contagion**	42	**32.312**	**8**	**50**	284.18	26	428	156.682	27	285
**Closed indirect contagion**	15	**11.14**	**1**	**20**	110.328	26	219	64.292	3	153
**Transitive contagion**	9	**3.654**	**0**	**9**	91.592	7	189	51.412	0	141
**Out-degree**	291	**270.964**	**217**	**347**	404.944	287	502	357.994	277	449
**In-degree**	289	**283.732**	**225**	**344**	389.432	277	502	382.184	269	484
**Reciprocal ties**	52	**46.45**	**31**	**62**	88.268	47	135	79.23	45	112
**In-two star**	228	**211.272**	**135**	**291**	456.796	203	682	407.524	187	580
**Out-two star**	286	**231.746**	**178**	**322**	403.704	269	556	346.052	253	466
**Mixed two-path**	306	**267.884**	**176**	**369**	615.826	263	878	548.166	303	833
**In-three star**	127	**154.726**	**60**	**258**	568.488	124	881	448.014	119	718
**Out-three star**	175	**110.108**	**77**	**162**	217.292	120	297	180.478	115	257
**Transitive triads**	87	**72.576**	**45**	**101**	179.81	103	290	142.16	76	229
**Cyclic**	32	**24.61**	**7**	**37**	111.516	37	196	85.1	31	156
**Indirect ties**	346	**291.322**	**218**	**381**	633.114	320	795	543.918	391	745
**Exclusively indirect ties**	244	**205.462**	**148**	**261**	405.982	263	539	362.93	244	452

Table S13 Goodness-of-fit results for models exploring *social influence* in the non-kin only *chatting* networks (the best fitting model is indicated in bold)

Parameter	Observed	Null			Direct			Reciprocal		
		Mean	HPDI		Mean	HPDI		Mean	HPDI	
			LI	UI		LI	UI		LI	UI
**Intercept**	325	343.59	296	386	315.19	280	350	324.808	290	361
**Direct contagion**	40	25.016	11	34	23.398	9	41	24.582	10	40
**Reciprocal contagion**	1	1.548	0	3	1.458	0	3	0.462	0	2
**Indirect contagion**	32	18.932	8	29	15.054	5	24	14.608	4	23
**Closed indirect contagion**	7	3.926	0	7	3.09	0	7	2.82	0	6
**Transitive contagion**	0	0.78	0	2	0.88	0	2	0.502	0	2
**Out-degree**	227	228.45	180	281	192.1	144	239	197.232	149	244
**In-degree**	195	214.504	174	265	180.63	140	219	177.482	142	215
**Reciprocal ties**	25	25.88	14	35	19.326	11	28	21.216	12	29
**In-two star**	101	130.91	74	190	104.17	59	146	96.226	53	130
**Out-two star**	206	190.654	141	240	156.378	108	203	161.576	96	210
**Mixed two-path**	204	197.212	137	259	150.73	98	208	147.766	96	202
**In-three star**	43	75.532	17	148	58.004	13	101	51.856	11	88
**Out-three star**	90	85.312	52	112	67.602	37	93	72.436	35	101
**Transitive triads**	33	31.552	20	45	24.704	10	35	24.052	12	33
**Cyclic**	9	5.75	1	10	3.732	0	7	3.49	0	6
**Indirect ties**	185	180.108	126	223	151.84	101	195	154.44	113	200
**Exclusively indirect ties**	147	141.776	106	182	121.534	84	157	125.302	91	163
**Parameter**	**Observed**	**Indirect**			**Closed indirect**			**Transitive**		
		**Mean**	**HPDI**		**Mean**	**HPDI**		**Mean**	**HPDI**	
			**LI**	**UI**		**LI**	**UI**		**LI**	**UI**
**Intercept**	325	318.19	290	340	**319.108**	**278**	**351**	296.434	268	322
**Direct contagion**	40	24.112	13	34	**41.33**	**22**	**58**	21.308	12	35
**Reciprocal contagion**	1	1.578	0	3	**3.284**	**1**	**6**	1.48	0	3
**Indirect contagion**	32	17.97	6	29	**19.82**	**5**	**32**	18.19	3	30
**Closed indirect contagion**	7	3.58	0	7	**3.486**	**0**	**7**	4.728	0	8
**Transitive contagion**	0	0.7	0	2	**1.386**	**0**	**4**	1.45	0	2
**Out-degree**	227	207.138	145	258	**234.332**	**180**	**286**	205.964	168	249
**In-degree**	195	185.734	147	217	**192.984**	**151**	**225**	164.898	126	198
**Reciprocal ties**	25	22.7	10	32	**24.562**	**15**	**35**	21.976	14	30
**In-two star**	101	114.126	69	167	**116.218**	**72**	**155**	96.312	57	135
**Out-two star**	206	174.106	95	231	**201.736**	**141**	**262**	175.956	130	228
**Mixed two-path**	204	193.946	94	257	**210.49**	**128**	**289**	173.762	112	236
**In-three star**	43	65.362	16	119	**67.12**	**24**	**114**	52.216	17	100
**Out-three star**	90	78.806	40	118	**91.202**	**52**	**120**	79.79	45	106
**Transitive triads**	33	27.494	9	39	**24.724**	**11**	**34**	27.87	11	40
**Cyclic**	9	4.672	0	8	**4.534**	**0**	**9**	5.55	0	10
**Indirect ties**	185	159.172	95	212	**177.012**	**114**	**245**	139.608	90	176
**Exclusively indirect ties**	147	125.912	76	167	**147.132**	**77**	**194**	106.596	72	134

Table S14 Goodness-of-fit results for models exploring *social influence* in the all alters *respect* networks (the best fitting model is indicated in bold)

**Parameter**	**Observed**	**Null**			**Direct**			**Reciprocal**		
		**Mean**	**HPDI**		**Mean**	**HPDI**		**Mean**	**HPDI**	
			**LI**	**UI**		**LI**	**UI**		**LI**	**UI**
**Intercept**	325	309.542	274	338	333.842	303	371	336.938	295	368
**Direct contagion**	69	47.470	27	69	48.926	26	67	72.892	32	117
**Reciprocal contagion**	3	1.064	0	3	0.926	0	2	1.542	0	3
**Indirect contagion**	112	87.350	42	135	85.12	36	127	132.75	44	211
**Closed indirect contagion**	25	14.434	2	25	13.71	3	22	27.586	4	53
**Transitive contagion**	11	1.944	0	5	1.902	0	5	6.12	0	14
**Out-degree**	453	452.992	378	511	473.24	411	532	497.624	378	597
**In-degree**	551	515.580	350	670	527.55	373	674	641.472	405	860
**Reciprocal ties**	23	17.218	9	24	17.07	8	23	23.234	13	34
**In-two star**	2905	3593.486	694	7057	3281.71	930	4641	5833.266	980	12302
**Out-two star**	490	496.180	397	562	515.358	440	588	544.08	395	661
**Mixed two-path**	916	978.734	591	1356	950.784	606	1396	1282.69	724	1943
**In-three star**	16698	55037.536	1707	87482	48852.79	1181	54567	109360.27	5631	317452
**Out-three star**	261	264.726	213	326	272.908	221	327	288.742	202	364
**Transitive triads**	145	142.618	107	174	148.368	114	173	161.922	114	204
**Cyclic**	16	14.048	6	20	14.364	7	22	18.754	6	29
**Indirect ties**	871	845.818	705	966	881.702	757	1019	924.986	681	1132
**Exclusively indirect ties**	660	642.480	551	742	670.67	561	756	698.724	505	846

**Parameter**	**Observed**	**Reciprocal and indirect**			**Reciprocal and closed indirect**			**Reciprocal and transitive**		
		**Mean**	**HPDI**		**Mean**	**HPDI**		**Mean**	**HPDI**	
			**LI**	**UI**		**LI**	**UI**		**LI**	**UI**
**Intercept**	325	327.27	293	358	**303.348**	**262**	**340**	298.284	256	337
**Direct contagion**	69	81.016	41	118	**66.53**	**31**	**104**	46.08	22	70
**Reciprocal contagion**	3	6.82	0	12	**2.018**	**0**	**4**	1.286	0	3
**Indirect contagion**	112	149.976	57	239	**105.676**	**33**	**178**	66.526	20	107
**Closed indirect contagion**	25	34.5	9	61	**29.454**	**6**	**59**	11.978	2	21
**Transitive contagion**	11	13.192	0	29	**6.614**	**0**	**15**	1.616	0	4
**Out-degree**	453	494.57	416	572	**426.668**	**344**	**526**	406.858	330	527
**In-degree**	551	608.35	427	812	**743.356**	**443**	**935**	614.976	402	861
**Reciprocal ties**	23	35.072	22	47	**16.578**	**7**	**25**	14.192	4	21
**In-two star**	2905	3902.978	1011	8071	**16980.502**	**1805**	**26364**	12372.356	1074	20663
**Out-two star**	490	547.432	434	633	**460.478**	**352**	**581**	436.492	338	573
**Mixed two-path**	916	1236.332	695	1728	**1049.234**	**585**	**1684**	775.72	495	1093
**In-three star**	16698	49387.208	2181	93992	**750189.5**	**4427**	**1187127**	560756.85	1158	946191
**Out-three star**	261	296.708	232	361	**242.054**	**177**	**327**	225.424	156	308
**Transitive triads**	145	160.434	125	197	**143.01**	**91**	**183**	127.844	89	174
**Cyclic**	16	28.512	17	40	**14.958**	**6**	**26**	12.332	4	21
**Indirect ties**	871	910.358	753	1062	**853.52**	**675**	**1069**	805.684	624	1040
**Exclusively indirect ties**	660	687.126	567	797	**651.544**	**518**	**805**	618.342	491	800

Table S15 Goodness-of-fit results for models exploring *social influence* in the kin only *respect* networks (the best fitting model is indicated in bold)

Parameter	Observed	Null			Direct			Reciprocal		
		Mean	HPDI		Mean	HPDI		Mean	HPDI	
			LI	UI		LI	UI		LI	UI
**Intercept**	325	300.996	266	332	325.464	287	361	355.504	324	387
**Direct contagion**	35	22.002	10	33	29.31	17	43	35.108	21	51
**Reciprocal contagion**	2	0.634	0	2	0.884	0	2	2.108	0	4
**Indirect contagion**	55	24.370	9	39	24.408	9	44	46.138	19	75
**Closed indirect contagion**	19	5.848	0	11	6.43	0	12	10.48	1	20
**Transitive contagion**	10	0.736	0	2	1.594	0	5	1.922	0	6
**Out-degree**	234	218.956	170	260	205.496	164	241	271.01	225	310
**In-degree**	305	268.158	199	342	250.508	183	310	352.882	243	449
**Reciprocal ties**	16	11.148	5	16	9.33	4	14	20.056	11	30
**In-two star**	851	796.622	282	1534	644.84	231	1085	1172.106	344	1966
**Out-two star**	192	181.326	138	223	160.784	109	203	226.886	176	271
**Mixed two-path**	324	292.700	165	421	234.276	110	360	421.294	217	624
**In-three star**	2572	4055.452	322	11894	2765.622	113	5478	6073.59	531	13703
**Out-three star**	91	83.026	57	109	70.84	38	95	103.44	72	129
**Transitive triads**	66	56.138	37	74	45.572	23	62	69.05	50	90
**Cyclic**	11	6.614	1	10	5.192	0	8	10.094	3	15
**Indirect ties**	283	246.136	164	300	224.906	154	278	309.498	245	380
**Exclusively indirect ties**	202	174.816	127	222	165.412	124	209	222.152	170	263

Parameter	Observed	Reciprocal and indirect			Reciprocal and closed indirect			Reciprocal and transitive		
		Mean	HPDI		Mean	HPDI		Mean	HPDI	
			LI	UI		LI	UI		LI	UI
**Intercept**	325	**350.392**	**317**	**373**	325.29	294	349	344.916	316	374
**Direct contagion**	35	**37.626**	**15**	**57**	28.052	12	40	36.396	9	68
**Reciprocal contagion**	2	**3.31**	**0**	**7**	2.016	0	4	0.822	0	3
**Indirect contagion**	55	**56.844**	**15**	**93**	37.466	11	64	70.088	10	144
**Closed indirect contagion**	19	**16.226**	**2**	**30**	6.956	0	14	18.29	0	43
**Transitive contagion**	10	**6.392**	**0**	**16**	1.602	0	5	6.444	0	23
**Out-degree**	234	**239.742**	**199**	**278**	268.936	228	308	250.426	192	318
**In-degree**	305	**348.972**	**262**	**433**	266.55	204	328	324.318	223	462
**Reciprocal ties**	16	**14.184**	**5**	**24**	12.99	4	19	20.938	11	29
**In-two star**	851	**1493.11**	**411**	**2280**	639.036	252	1072	1066.108	194	2003
**Out-two star**	192	**193.086**	**152**	**238**	231.428	180	271	199.276	119	267
**Mixed two-path**	324	**473.39**	**244**	**671**	314.856	154	422	341.438	155	548
**In-three star**	2572	**10327.724**	**875**	**18771**	2268.308	279	5178	5284.884	193	12576
**Out-three star**	91	**86.41**	**61**	**115**	108.176	75	135	88.028	40	125

**Transitive triads**	66	**63.396**	**38**	**84**	66.012	40	89	63.044	22	96
**Cyclic**	11	**10.938**	**1**	**19**	7.59	2	12	10.384	4	17
**Indirect ties**	283	**278.562**	**204**	**359**	321.22	260	390	291.384	159	412
**Exclusively indirect ties**	202	**198.266**	**152**	**255**	236.978	181	284	209.476	135	292

Table S16 Goodness-of-fit results for models exploring *social influence* in the non-kin only *respect* networks (the best fitting model is indicated in bold)

Parameter	Observed	Null			Direct			Reciprocal		
		Mean	HPDI		Mean	HPDI		Mean	HPDI	
			LI	UI		LI	UI		LI	UI
**Intercept**	325	299.990	269	327	**321.432**	**285**	**355**	301.912	261	340
**Direct contagion**	34	20.262	7	30	**26.002**	**9**	**40**	15.71	3	25
**Reciprocal contagion**	1	0.216	0	1	**0.352**	**0**	**1**	0.202	0	1
**Indirect contagion**	20	20.248	5	34	**22.18**	**4**	**39**	12.916	2	23
**Closed indirect contagion**	1	1.684	0	4	**2.484**	**0**	**6**	1.158	0	3
**Transitive contagion**	0	0.276	0	1	**0.408**	**0**	**2**	0.162	0	1
**Out-degree**	219	208.182	168	247	**201.1**	**145**	**254**	202.824	146	262
**In-degree**	246	221.250	144	289	**251.678**	**141**	**362**	169.778	112	240
**Reciprocal ties**	6	3.608	1	7	**3.802**	**0**	**6**	4.824	2	8
**In-two star**	666	1066.116	179	1748	**1435.818**	**144**	**3713**	477.45	83	863
**Out-two star**	179	159.664	116	196	**149.73**	**88**	**202**	150.786	97	211
**Mixed two-path**	175	240.834	98	368	**228.41**	**80**	**363**	164.038	54	263
**In-three star**	1956	11875.252	264	13803	**18189.822**	**106**	**75027**	2580.676	40	5581
**Out-three star**	74	63.656	41	85	**58.726**	**27**	**85**	58.784	33	92
**Transitive triads**	18	22.360	11	32	**20.164**	**10**	**31**	20.244	8	29
**Cyclic**	0	1.510	0	3	**1.162**	**0**	**2**	1.37	0	3
**Indirect ties**	183	200.020	153	244	**194.084**	**131**	**256**	191.602	127	252
**Exclusively indirect ties**	161	169.490	134	208	**166.204**	**102**	**208**	163.952	116	218
**Parameter**	**Observed**	**Reciprocal and indirect**			**Reciprocal and closed indirect**			**Reciprocal and transitive**		
		**Mean**	**HPDI**		**Mean**	**HPDI**		**Mean**	**HPDI**	
			**LI**	**UI**		**LI**	**UI**		**LI**	**UI**
**Intercept**	325	310.544	279	343	293.158	254	323	304.332	272	333
**Direct contagion**	34	40.86	16	67	19.556	6	32	23.662	12	33
**Reciprocal contagion**	1	0.684	0	2	0.518	0	2	1.088	0	3
**Indirect contagion**	20	25.086	7	43	14.176	3	26	15.364	4	26
**Closed indirect contagion**	1	3.956	0	10	0.324	0	1	1.39	0	3
**Transitive contagion**	0	1.138	0	3	0.04	0	0	0.124	0	0
**Out-degree**	219	245.788	189	298	178.366	144	204	207.506	176	247
**In-degree**	246	264.57	148	375	222.352	123	308	206.382	140	267
**Reciprocal ties**	6	5.744	3	9	3.93	0	8	8.06	2	12
**In-two star**	666	1407.594	216	4118	1360.648	114	3284	658.106	169	1206
**Out-two star**	179	197.338	142	252	124.082	93	151	157.57	121	190
**Mixed two-path**	175	208.334	96	320	156.81	59	278	169.758	79	241
**In-three star**	1956	18877.54	225	76787	19316.21	335	33516	4137.046	87	7502
**Out-three star**	74	81.756	43	107	44.834	27	61	62.194	43	83
**Transitive triads**	18	27.55	15	40	9.416	4	15	17.214	9	23

**Cyclic**	0	1.782	0	4	0.914	0	2	1.652	0	3
**Indirect ties**	183	242.87	170	320	141.882	106	172	164.202	123	199
**Exclusively indirect ties**	161	204.826	141	263	128.678	97	157	141.128	105	173

Table S17 Goodness-of-fit results for models exploring *social influence* in the all alters *money* networks (full sample, the best fitting model is indicated in bold)

Parameter	Observed	Null			Direct			Reciprocal		
		Mean	HPDI		Mean	HPDI		Mean	HPDI	
			LI	UI		LI	UI		LI	UI
Intercept	325	322.204	284	355	317.2	277	350	**333.312**	**291**	**372**
Direct contagion	71	47.214	31	66	57.134	37	78	**68.744**	**38**	**95**
Reciprocal contagion	16	9.398	4	15	12.448	6	19	**12.688**	**5**	**19**
Indirect contagion	231	147.526	87	190	135.102	75	189	**189.564**	**115**	**256**
Closed indirect contagion	34	13.494	3	22	15.146	2	25	**20.57**	**3**	**32**
Transitive contagion	6	1.282	0	4	3.012	0	9	**3.64**	**0**	**9**
Out-degree	538	516.75	427	594	500.944	399	576	**575.938**	**486**	**650**
In-degree	576	572.046	489	660	541.812	444	636	**617.366**	**518**	**693**
Reciprocal ties	239	225.608	186	269	211.06	167	255	**245.582**	**201**	**288**
In-two star	743	702.74	557	845	642.464	459	804	**794.298**	**633**	**942**
Out-two star	769	705.716	505	885	663.462	486	859	**890.12**	**619**	**1158**
Mixed two-path	1527	1452.452	1109	1733	1352.666	935	1655	**1715.886**	**1324**	**2057**
In-three star	696	669.634	445	853	594.964	336	843	**816.916**	**508**	**1049**
Out-three star	927	920.456	437	1428	811.408	437	1195	**1709.818**	**550**	**2752**
Transitive triads	169	156.51	111	193	141.776	103	186	**175.874**	**128**	**216**
Cyclic	185	149.66	110	181	131.462	88	163	**164.37**	**129**	**202**
Indirect ties	1624	1594.988	1321	1879	1508.702	1139	1817	**1755.182**	**1425**	**2046**
Exclusively indirect ties	1362	1348.838	1084	1551	1285.542	948	1537	**1481.5**	**1251**	**1774**

Parameter	Observed	Reciprocal and indirect			Reciprocal and closed indirect			Reciprocal and transitive		
		Mean	HPDI		Mean	HPDI		Mean	HPDI	
			LI	UI		LI	UI		LI	UI
Intercept	325	338.184	267	402	347.708	297	385	345.484	309	383
Direct contagion	71	147.958	38	340	102.244	34	182	56.82	28	85
Reciprocal contagion	16	44.218	5	109	21.222	5	39	11.954	4	20
Indirect contagion	231	653.366	97	1685	485.516	139	993	170.534	86	242
Closed indirect contagion	34	97.612	7	235	29.506	3	60	21.418	5	35
Transitive contagion	6	61.834	0	177	8.48	0	23	1.324	0	4
Out-degree	538	704.498	457	1011	612.812	469	777	563.968	434	684
In-degree	576	722.828	515	1021	667.118	520	826	577.898	468	685
Reciprocal ties	239	330.458	167	505	269.982	196	335	238.144	172	302
In-two star	743	1066.23	637	1705	893.252	587	1255	782.306	513	1043
Out-two star	769	1330.192	556	2007	935.46	480	1321	1499.072	364	2004
Mixed two-path	1527	2390.548	1257	3806	1881.326	1155	2719	1989.58	1023	2723
In-three star	696	1249.768	586	2150	957.73	432	1540	1054.194	377	1531
Out-three star	927	3579.392	580	10062	1714.774	366	2213	11904.714	277	12653
Transitive triads	169	268.172	121	429	164.234	107	220	189.264	111	259
Cyclic	185	244.12	115	385	156.626	105	195	166.264	104	215
Indirect ties	1624	2439.342	1294	4106	2309.358	1468	3119	1553.562	1086	1954
Exclusively indirect ties	1362	2021.05	1137	3453	2033.674	1292	2782	1268.06	915	1589

Table S18 Goodness-of-fit results for models exploring *social influence* in the kin only *money* networks (full sample, the best fitting model is indicated in bold)

**Parameter**	**Observed**	**Null**			**Direct**			**Reciprocal**		
		**Mean**	**HPDI**		**Mean**	**HPDI**		**Mean**	**HPDI**	
			**LI**	**UI**		**LI**	**UI**		**LI**	**UI**
**Intercept**	325	334.672	310	362	**328.588**	**303**	**361**	318.462	285	356
**Direct contagion**	44	25.494	14	36	**36.58**	**21**	**50**	33.568	16	51
**Reciprocal contagion**	5	2.092	0	4	**3.906**	**0**	**7**	3.058	0	6
**Indirect contagion**	42	30.436	12	45	**38.918**	**16**	**60**	33.59	11	49
**Closed indirect contagion**	13	6.668	1	12	**11.588**	**2**	**20**	8.81	1	16
**Transitive contagion**	5	0.778	0	2	**3.054**	**0**	**7**	2.48	0	6
**Out-degree**	242	231.03	181	263	**261.29**	**210**	**313**	235.162	194	281
**In-degree**	302	308.416	261	351	**310.27**	**262**	**363**	280.142	233	318
**Reciprocal ties**	43	41.754	27	56	**47.31**	**31**	**63**	39.524	23	50
**In-two star**	294	263.636	201	313	**268.476**	**199**	**324**	234.922	185	276
**Out-two star**	215	193.774	113	279	**262.688**	**160**	**371**	232.502	122	317
**Mixed two-path**	311	291.098	200	366	**340.914**	**217**	**439**	294.526	202	386
**In-three star**	153	126.234	82	159	**128.562**	**88**	**173**	109.388	72	139
**Out-three star**	218	209.318	43	476	**326.8**	**69**	**604**	289.616	74	543
**Transitive triads**	46	55.238	32	72	**69.924**	**40**	**95**	55.938	34	76
**Cyclic**	19	27.038	12	39	**30.266**	**14**	**45**	23.666	8	34
**Indirect ties**	232	257.088	174	309	**292.392**	**197**	**359**	257.906	187	325
**Exclusively indirect ties**	178	189.52	135	234	**207.474**	**148**	**253**	189.598	137	235

**Parameter**	**Observed**	**Indirect**			**Closed indirect**			**Transitive**		
		**Mean**	**HPDI**		**Mean**	**HPDI**		**Mean**	**HPDI**	
			**LI**	**UI**		**LI**	**UI**		**LI**	**UI**
**Intercept**	325	315.23	282	347	317.492	287	344	330.342	290	371
**Direct contagion**	44	24.428	13	36	51.456	18	84	53.058	31	78
**Reciprocal contagion**	5	2.198	0	4	9.514	0	20	6.444	1	10
**Indirect contagion**	42	30.734	10	51	68.098	15	132	40.186	18	62
**Closed indirect contagion**	13	7.286	0	15	33.092	1	81	12.628	3	22
**Transitive contagion**	5	1.262	0	4	23.27	0	67	0.906	0	3
**Out-degree**	242	242.256	185	284	291.078	234	355	256.948	202	324
**In-degree**	302	310.746	251	366	302.522	250	351	347.834	271	426
**Reciprocal ties**	43	44.712	31	57	57.626	33	82	52.826	33	72
**In-two star**	294	274.758	210	338	269.626	188	331	311.884	232	399
**Out-two star**	215	232.192	106	368	354.996	176	567	262.326	135	380
**Mixed two-path**	311	331.33	225	445	458.91	273	681	398.914	246	558
**In-three star**	153	134.4	92	178	130.53	78	179	152.5	92	197
**Out-three star**	218	290.048	34	649	532.614	119	1115	337.882	64	638
**Transitive triads**	46	59.784	25	91	85.256	28	147	71.92	34	110
**Cyclic**	19	26.692	9	43	46.686	13	90	35.314	15	54
**Indirect ties**	232	244.254	145	341	355.28	221	538	282.856	183	373
**Exclusively indirect ties**	178	172.442	112	234	251.442	163	340	194.382	139	256

Table S19 Goodness-of-fit results for models exploring *social influence* in the non-kin only *money* networks (full sample, the best fitting model is indicated in bold)

Parameter	Observed	Null			Direct			Reciprocal		
		Mean	HPDI		Mean	HPDI		Mean	HPDI	
			LI	UI		LI	UI		LI	UI
**Intercept**	325	322.86	296	357	344.78	308	381	318.988	291	346
**Direct contagion**	25	12.512	5	19	20.52	9	32	20.812	11	31
**Reciprocal contagion**	3	0.56	0	2	0.996	0	2	3.044	0	6
**Indirect contagion**	28	9.776	1	15	12.826	4	21	14.268	5	23
**Closed indirect contagion**	4	1.108	0	3	1.688	0	4	2.81	0	5
**Transitive contagion**	0	0.078	0	0	0.318	0	1	0.63	0	3
**Out-degree**	146	136.466	111	167	134.268	110	166	153.146	126	185
**In-degree**	184	176.56	134	210	210.278	165	270	189.094	148	225
**Reciprocal ties**	28	14.3	7	20	16.182	7	23	34.032	24	45
**In-two star**	154	123.484	88	159	154.144	106	202	143.878	102	184
**Out-two star**	74	74.462	29	119	66.748	28	88	93.022	38	138
**Mixed two-path**	164	108.21	57	147	119.928	67	168	160.08	102	217
**In-three star**	73	48.416	25	66	61.682	33	85	61.616	36	87
**Out-three star**	34	56.246	4	120	62.57	5	68	69.27	7	139
**Transitive triads**	19	11.236	3	18	10.154	5	18	16.688	8	24
**Cyclic**	7	2.934	0	6	3.394	0	6	7.458	2	11
**Indirect ties**	115	100.514	65	134	96.256	58	126	122.986	83	163
**Exclusively indirect ties**	94	87.148	60	117	84.098	52	109	103.492	70	140
**Parameter**	**Observed**	**Reciprocal and indirect**			**Reciprocal and closed indirect**			**Reciprocal and transitive**		
		**Mean**	**HPDI**		**Mean**	**HPDI**		**Mean**	**HPDI**	
			**LI**	**UI**		**LI**	**UI**		**LI**	**UI**
**Intercept**	325	360.106	308	408	334.768	301	367	**311.104**	**279**	**335**
**Direct contagion**	25	43.506	21	75	16.498	7	26	**19.526**	**5**	**30**
**Reciprocal contagion**	3	5.83	0	13	1.532	0	3	**3.868**	**0**	**7**
**Indirect contagion**	28	32.656	5	58	12.906	5	21	**23.256**	**6**	**40**
**Closed indirect contagion**	4	8.608	0	20	2.96	0	6	**6.614**	**0**	**14**
**Transitive contagion**	0	5.948	0	16	0.584	0	2	**2.642**	**0**	**9**
**Out-degree**	146	165.168	128	202	157.912	117	194	**138.748**	**105**	**168**
**In-degree**	184	193.44	158	235	180.828	139	214	**176.672**	**143**	**210**
**Reciprocal ties**	28	34.666	18	47	26.648	17	35	**33.266**	**20**	**43**
**In-two star**	154	144.006	108	186	128.256	81	161	**141.06**	**106**	**178**
**Out-two star**	74	112.874	46	158	120.416	50	202	**151.566**	**51**	**237**
**Mixed two-path**	164	175.676	115	246	149.912	78	206	**210.1**	**131**	**286**
**In-three star**	73	60.176	38	83	52.098	28	74	**64.956**	**41**	**89**
**Out-three star**	34	195.816	9	162	212.16	21	610	**362.764**	**16**	**648**

**Transitive triads**	19	18.68	7	29	19.832	11	30	**27.044**	**10**	**40**
**Cyclic**	7	8.326	1	14	7.06	3	11	**11.208**	**4**	**17**
**Indirect ties**	115	116.476	75	162	120.43	86	152	**136.668**	**79**	**196**
**Exclusively indirect ties**	94	95.486	61	133	97.39	69	126	**105.442**	**61**	**155**

Table S20 Full results from the best fitting ALAAM models exploring contagion, i.e. *social influence*, in the estimated kin and non-kin only *chatting* networks

Parameter	Mean	sd	Credible interval	
			**0.025**	**0.975**
Kin only alters				
**Intercept**	-1.775	0.322	-2.413	-1.161
**Direct contagion**	**0.412**	**0.234**	**-0.045**	**0.852**
**Indirect contagion**	**0.052**	**0.197**	**-0.344**	**0.413**
**Gender**	-0.041	0.211	-0.43	0.393
**Age**	0.017	0.006	0.005	0.028
**Some primary vs none**	-0.381	0.244	-0.856	0.113
**Completed primary vs none**	-0.171	0.388	-0.952	0.559
**Some secondary vs none**	-0.685	0.386	-1.48	0.014
**Zone 2 vs 1**	-0.243	0.335	-0.9	0.424
**Zone 3 vs 1**	-0.432	0.371	-1.194	0.255
**Zone 4 vs 1**	-0.49	0.392	-1.312	0.208
**Zone 5 vs 1**	-0.504	0.28	-1.085	0.021
**Zone 6 vs 1**	-1.119	0.476	-2.094	-0.267
**Zone 7 vs 1**	-1.683	0.421	-2.501	-0.864
**Zone 8 vs 1**	-1.696	0.351	-2.378	-1.018
**Zone 9 vs 1**	-2.802	1.164	-5.302	-0.894
**Out-degree**	-0.007	0.125	-0.253	0.222
**In-degree**	-0.065	0.104	-0.291	0.127
**Brokerage**	-0.06	0.059	-0.182	0.043
**Indirect**	-0.003	0.068	-0.14	0.13
Non-kin only alters				
**Intercept**	-1.865	0.311	-2.538	-1.298
**Direct contagion**	**0.623**	**0.276**	**0.092**	**1.166**
**Indirect contagion**	**0.236**	**0.275**	**-0.315**	**0.765**
**Closed indirect contagion**	**-0.533**	**0.682**	**-1.93**	**0.748**
**Gender**	-0.012	0.215	-0.44	0.396
**Age**	0.017	0.006	0.005	0.029
**Some primary vs none**	-0.382	0.249	-0.847	0.098
**Completed primary vs none**	-0.163	0.38	-0.895	0.602
**Some secondary vs none**	-0.677	0.414	-1.446	0.137
**Zone 2 vs 1**	-0.233	0.33	-0.88	0.4
**Zone 3 vs 1**	-0.3	0.339	-0.992	0.341
**Zone 4 vs 1**	-0.401	0.374	-1.195	0.254
**Zone 5 vs 1**	-0.527	0.297	-1.17	0.007
**Zone 6 vs 1**	-1.11	0.534	-2.311	-0.176
**Zone 7 vs 1**	-1.58	0.38	-2.343	-0.854
**Zone 8 vs 1**	-1.559	0.344	-2.285	-0.924
**Zone 9 vs 1**	-2.993	1.283	-6.139	-1.001
**Out-degree**	-0.026	0.136	-0.297	0.231
**In-degree**	-0.221	0.134	-0.491	0.045
**Brokerage**	0.055	0.076	-0.095	0.196
**Indirect**	0.01	0.256	-0.588	0.438
**Indirect exclusive**	-0.068	0.292	-0.594	0.562

Table S21 Full results from the best fitting ALAAM models exploring contagion, i.e. *social influence*, in the estimated *respect* networks

Parameter	Mean	sd	Credible interval	
			**0.025**	**0.975**
All alters				
Intercept	-2.366	0.416	-3.234	-1.562
**Direct contagion**	**0.348**	**0.198**	**-0.015**	**0.733**
**Reciprocal contagion**	**-0.204**	**1.321**	**-3.031**	**2.198**
**Indirect contagion**	**-0.012**	**0.093**	**-0.213**	**0.161**
**Closed indirect contagion**	**-0.036**	**0.318**	**-0.731**	**0.558**
Gender	-0.01	0.202	-0.413	0.383
Age	0.018	0.007	0.004	0.03
Some primary vs none	-0.356	0.247	-0.844	0.127
Completed primary vs none	-0.177	0.372	-0.911	0.527
Some secondary vs none	-0.769	0.395	-1.601	-0.01
Zone 2 vs 1	-0.388	0.304	-0.96	0.23
Zone 3 vs 1	-0.466	0.34	-1.108	0.186
Zone 4 vs 1	-0.48	0.351	-1.189	0.202
Zone 5 vs 1	-0.607	0.306	-1.261	-0.058
Zone 6 vs 1	-1.267	0.521	-2.377	-0.357
Zone 7 vs 1	-1.608	0.388	-2.441	-0.919
Zone 8 vs 1	-1.77	0.361	-2.567	-1.083
Zone 9 vs 1	-2.745	1.103	-5.413	-0.98
Out-degree	-0.093	0.126	-0.362	0.134
In-degree	-0.045	0.047	-0.15	0.03
Reciprocation	0.295	0.479	-0.837	1.146
Brokerage	-0.017	0.019	-0.055	0.023
Indirect	0.015	0.108	-0.219	0.226
Indirect exclusive	0.022	0.132	-0.246	0.284
Kin only alters				
Intercept	-2.279	0.417	-3.108	-1.481
**Direct contagion**	**0.018**	**0.271**	**-0.498**	**0.549**
**Reciprocal contagion**	**-0.686**	**1.812**	**-4.62**	**2.495**
**Indirect contagion**	**0.269**	**0.171**	**-0.086**	**0.603**
Gender	-0.084	0.208	-0.498	0.331
Age	0.017	0.006	0.004	0.029
Some primary vs none	-0.374	0.256	-0.854	0.156
Completed primary vs none	-0.203	0.374	-0.987	0.491
Some secondary vs none	-0.741	0.387	-1.538	-0.001
Zone 2 vs 1	-0.308	0.316	-0.905	0.289
Zone 3 vs 1	-0.468	0.439	-1.406	0.34
Zone 4 vs 1	-0.62	0.355	-1.351	0.02
Zone 5 vs 1	-0.551	0.281	-1.126	-0.001
Zone 6 vs 1	-1.246	0.52	-2.357	-0.301
Zone 7 vs 1	-1.604	0.414	-2.489	-0.84
Zone 8 vs 1	-1.764	0.381	-2.545	-1.054
Zone 9 vs 1	-2.738	1.318	-6.249	-0.883
Out-degree	-0.066	0.133	-0.345	0.179
In-degree	-0.07	0.071	-0.217	0.058
Reciprocation	0.252	0.634	-1.12	1.402
Brokerage	-0.026	0.038	-0.107	0.044
Indirect	0	0.08	-0.148	0.168

Table S22 Full results from the best fitting ALAAM models exploring contagion, i.e. *social influence*, in the estimated *money* networks (full sample)

Parameter	Mean	sd	Credible interval	
			**0.025**	**0.975**
All alters				
Intercept	-1.609	0.293	-2.189	-1.057
**Direct contagion**	**0.321**	**0.224**	**-0.151**	**0.741**
**Reciprocal contagion**	**-0.255**	**0.637**	**-1.54**	**0.959**
Gender	-0.078	0.199	-0.477	0.285
Age	0.016	0.006	0.004	0.028
Some primary vs none	-0.409	0.257	-0.885	0.094
Completed primary vs none	-0.239	0.361	-0.946	0.476
Some secondary vs none	-0.734	0.396	-1.537	-0.002
Zone 2 vs 1	-0.297	0.314	-0.898	0.319
Zone 3 vs 1	-0.495	0.367	-1.254	0.148
Zone 4 vs 1	-0.512	0.364	-1.26	0.162
Zone 5 vs 1	-0.637	0.283	-1.229	-0.103
Zone 6 vs 1	-1.23	0.5	-2.293	-0.337
Zone 7 vs 1	-1.577	0.391	-2.331	-0.83
Zone 8 vs 1	-1.767	0.342	-2.484	-1.146
Zone 9 vs 1	-3.002	1.185	-5.704	-1.147
Out-degree	-0.143	0.086	-0.32	0.017
In-degree	-0.012	0.089	-0.195	0.157
Reciprocation	0.075	0.141	-0.197	0.349
Non-kin only				
Intercept	-2.334	0.439	-3.167	-1.451
**Direct contagion**	**0.53**	**0.37**	**-0.234**	**1.228**
**Reciprocal contagion**	**-0.779**	**1.303**	**-3.498**	**1.606**
Gender	-0.025	0.209	-0.466	0.363
Age	0.018	0.006	0.004	0.03
Some primary vs none	-0.384	0.268	-0.907	0.148
Completed primary vs none	-0.151	0.406	-1.101	0.553
Some secondary vs none	-0.664	0.391	-1.462	0.061
Zone 2 vs 1	-0.354	0.31	-0.946	0.261
Zone 3 vs 1	-0.389	0.351	-1.102	0.283
Zone 4 vs 1	-0.488	0.38	-1.319	0.188
Zone 5 vs 1	-0.631	0.286	-1.17	-0.086
Zone 6 vs 1	-1.287	0.54	-2.421	-0.328
Zone 7 vs 1	-1.626	0.436	-2.521	-0.831
Zone 8 vs 1	-1.8	0.361	-2.554	-1.126
Zone 9 vs 1	-2.84	1.404	-6.491	-0.884
Out-degree	-0.263	0.132	-0.545	-0.023
In-degree	-0.091	0.104	-0.318	0.108
Reciprocation	0.807	0.415	-0.066	1.58

Table S23 Full results from the STRAND kebele-zone 1 model exploring *social selection* in the latent *marriage-advice* networks

Variable		Median	LI	HI	Mean	SD
**Focal (out-degree) effects**	Age	-0.533	-1.067	-0.067	-0.008	0.005
	Some primary vs. none	-0.308	-0.805	0.195	-0.160	0.156
	Complete primary vs. none	-0.530	-1.072	-0.072	-0.464	0.260
	Some secondary vs. none	-0.146	-0.650	0.350	-0.092	0.196
	Community role vs. none	0.272	-0.266	0.734	0.185	0.220
**Target (in-degree) effects**	Age	1.938	1.500	2.500	0.031	0.005
	Some primary vs. none	0.013	-0.414	0.586	0.012	0.158
	Complete primary vs. none	0.224	-0.285	0.715	0.190	0.264
	Some secondary vs. none	-0.275	-0.788	0.212	-0.212	0.229
	Community role vs. none	-0.291	-0.767	0.233	-0.220	0.233
**Dyadic (network overlap) effects**	Chatting	8.122	7.616	8.616	6.865	0.261
	Respect	6.741	6.269	7.269	4.718	0.217
	Money-borrowing	8.179	7.665	8.665	6.094	0.231
**Block (offset) effects**	Any to any	1.566	-0.823	3.953	1.581	1.449
	Anti- to anti-FGMC	-4.182	-5.951	-2.426	-4.202	1.087
	Anti- to pro-FGMC	-4.385	-6.207	-2.669	-4.403	1.091
	Pro- to anti-FGMC	-4.091	-5.864	-2.339	-4.109	1.095
	Pro- to pro-FGMC	-4.497	-6.491	-2.759	-4.517	1.149
	Woman to woman	-4.800	-6.605	-2.862	-4.754	1.150
	Woman to man	-3.845	-5.570	-1.812	-3.796	1.149
	Man to woman	-5.677	-7.420	-3.669	-5.618	1.151
	Man to man	-4.075	-5.734	-1.968	-4.023	1.150
**Focal effects SD**		0.233	0.001	0.420	0.237	0.138
**Target effects SD**		0.449	0.279	0.627	0.447	0.107
**Dyadic effects SD**		0.437	0.003	0.923	0.475	0.317
**Focal-target effects rho**		0.397	-0.233	0.888	0.336	0.359
**Dyadic effects rho**		0.034	-0.578	0.695	0.027	0.386

Table S24 Full results from the STRAND kebele-zone 2 model exploring *social selection* in the latent *marriage-advice* networks

Variable		Median	LI	HI	Mean	SD
**Focal (out-degree) effects**	Age	-0.583	-1.083	-0.083	-0.014	0.008
	Some primary vs. none	0.045	-0.458	0.542	0.035	0.257
	Complete primary vs. none	0.148	-0.335	0.665	0.185	0.388
	Some secondary vs. none	0.062	-0.424	0.576	0.068	0.350
	Community role vs. none	0.141	-0.365	0.635	0.150	0.354
**Target (in-degree) effects**	Age	1.400	0.880	1.880	0.035	0.008
	Some primary vs. none	-0.120	-0.605	0.395	-0.109	0.275
	Complete primary vs. none	-0.037	-0.523	0.477	-0.063	0.517
	Some secondary vs. none	0.039	-0.471	0.529	0.049	0.365
	Community role vs. none	0.075	-0.449	0.551	0.074	0.400
**Dyadic (network overlap) effects**	Chatting	4.776	4.238	5.238	5.914	0.376
	Respect	4.990	4.483	5.483	4.971	0.300
	Money-borrowing	3.646	3.155	4.155	4.916	0.412
**Block (offset) effects**	Any to any	1.325	-1.174	3.653	1.340	1.459
	Anti- to anti-FGMC	-3.833	-5.696	-1.915	-3.815	1.158
	Anti- to pro-FGMC	-4.899	-6.927	-3.014	-4.876	1.195
	Pro- to anti-FGMC	-3.846	-5.733	-1.897	-3.824	1.180
	Pro- to pro-FGMC	-3.963	-6.173	-1.842	-3.974	1.316
	Woman to woman	-4.958	-6.823	-3.009	-4.977	1.163
	Woman to man	-3.375	-5.196	-1.476	-3.392	1.132
	Man to woman	-4.495	-6.331	-2.573	-4.498	1.153
	Man to man	-3.911	-5.814	-2.078	-3.926	1.140
**Focal effects SD**		0.237	0.000	0.535	0.268	0.192
**Target effects SD**		0.456	0.025	0.755	0.450	0.224
**Dyadic effects SD**		0.249	0.000	0.689	0.319	0.265
**Focal-target effects rho**		-0.087	-0.718	0.562	-0.061	0.394
**Dyadic effects rho**		0.043	-0.654	0.679	0.030	0.408

Table S25 Full results from the STRAND kebele-zone 3 model exploring *social selection* in the latent *marriage-advice* networks

Variable		Median	LI	HI	Mean	SD
**Focal (out-degree) effects**	Age	0.174	-0.348	0.652	0.004	0.007
	Some primary vs. none	-0.106	-0.591	0.409	-0.082	0.255
	Complete primary vs. none	0.274	-0.203	0.797	0.313	0.349
	Some secondary vs. none	0.003	-0.511	0.489	0.005	0.378
	Community role vs. none	-0.492	-0.993	0.007	-0.547	0.334
**Target (in-degree) effects**	Age	1.516	1.000	2.000	0.047	0.009
	Some primary vs. none	0.453	-0.030	0.970	0.525	0.353
	Complete primary vs. none	0.445	-0.091	0.909	0.755	0.518
	Some secondary vs. none	0.485	-0.021	0.979	0.778	0.499
	Community role vs. none	-0.496	-1.002	-0.002	-0.672	0.410
**Dyadic (network overlap) effects**	Chatting	3.704	3.222	4.222	4.659	0.380
	Respect	3.259	2.787	3.787	3.571	0.337
	Money-borrowing	3.003	2.469	3.469	3.840	0.390
**Block (offset) effects**	Any to any	1.116	-1.386	3.427	1.109	1.474
	Anti- to anti-FGMC	-4.153	-6.107	-2.302	-4.151	1.161
	Anti- to pro-FGMC	-4.002	-5.923	-2.068	-4.019	1.177
	Pro- to anti-FGMC	-4.282	-6.198	-2.296	-4.271	1.194
	Pro- to pro-FGMC	-3.455	-5.477	-1.427	-3.461	1.230
	Woman to woman	-5.072	-7.144	-3.225	-5.062	1.200
	Woman to man	-3.203	-5.035	-1.196	-3.197	1.171
	Man to woman	-4.949	-6.892	-2.942	-4.951	1.198
	Man to man	-3.299	-5.262	-1.398	-3.284	1.165
**Focal effects SD**		0.158	0.000	0.377	0.185	0.137
**Target effects SD**		1.001	0.678	1.324	1.014	0.201
**Dyadic effects SD**		0.617	0.000	1.218	0.655	0.413
**Focal-target effects rho**		0.192	-0.477	0.789	0.154	0.390
**Dyadic effects rho**		0.160	-0.528	0.766	0.122	0.400

Table S26 Full results from the STRAND kebele-zone 4 model exploring *social selection* in the latent *marriage-advice* networks

Variable		Median	LI	HI	Mean	SD
**Focal (out-degree) effects**	Age	-0.167	-0.667	0.333	-0.004	0.007
	Some primary vs. none	0.088	-0.412	0.588	0.090	0.305
	Complete primary vs. none	0.230	-0.252	0.748	0.285	0.375
	Some secondary vs. none	-0.313	-0.813	0.187	-0.440	0.424
	Community role vs. none	-0.029	-0.524	0.476	-0.045	0.401
**Target (in-degree) effects**	Age	1.037	0.556	1.556	0.028	0.009
	Some primary vs. none	-0.365	-0.874	0.126	-0.417	0.347
	Complete primary vs. none	0.001	-0.484	0.516	0.002	0.476
	Some secondary vs. none	-0.058	-0.574	0.426	-0.082	0.423
	Community role vs. none	-0.390	-0.886	0.114	-0.727	0.562
**Dyadic (network overlap) effects**	Chatting	3.978	3.475	4.475	5.381	0.413
	Respect	2.660	2.174	3.174	3.351	0.382
	Money-borrowing	3.213	2.725	3.725	4.386	0.420
**Block (offset) effects**	Any to any	1.047	-1.489	3.527	1.048	1.547
	Anti- to anti-FGMC	-3.001	-5.004	-0.964	-3.009	1.241
	Anti- to pro-FGMC	-5.361	-7.765	-3.144	-5.391	1.401
	Pro- to anti-FGMC	-2.528	-4.567	-0.394	-2.531	1.257
	Pro- to pro-FGMC	-4.775	-8.049	-1.803	-4.888	1.911
	Woman to woman	-4.565	-6.533	-2.621	-4.544	1.192
	Woman to man	-3.295	-5.238	-1.315	-3.283	1.188
	Man to woman	-4.924	-6.908	-2.829	-4.929	1.244
	Man to man	-3.857	-5.752	-1.821	-3.844	1.193
**Focal effects SD**		0.153	0.000	0.374	0.181	0.138
**Target effects SD**		0.727	0.390	1.074	0.726	0.212
**Dyadic effects SD**		0.372	0.000	0.923	0.440	0.334
**Focal-target effects rho**		0.066	-0.621	0.724	0.054	0.412
**Dyadic effects rho**		-0.011	-0.680	0.654	-0.006	0.411

Table S27 Full results from the STRAND kebele-zone 5 model exploring *social selection* in the latent *marriage-advice* networks

Variable		Median	LI	HI	Mean	SD
**Focal (out-degree) effects**	Age	-0.550	-1.050	-0.050	-0.011	0.006
	Some primary vs. none	0.303	-0.176	0.824	0.244	0.240
	Complete primary vs. none	0.228	-0.295	0.705	0.253	0.342
	Some secondary vs. none	-0.030	-0.523	0.477	-0.036	0.349
	Community role vs. none	0.213	-0.276	0.724	0.200	0.296
**Target (in-degree) effects**	Age	1.320	0.840	1.840	0.033	0.008
	Some primary vs. none	-0.168	-0.682	0.318	-0.155	0.279
	Complete primary vs. none	-0.285	-0.766	0.234	-0.422	0.448
	Some secondary vs. none	0.021	-0.472	0.528	0.022	0.396
	Community role vs. none	-0.429	-0.930	0.070	-0.531	0.379
**Dyadic (network overlap) effects**	Chatting	6.404	5.917	6.917	6.957	0.331
	Respect	5.872	5.383	6.383	5.310	0.275
	Money-borrowing	6.286	5.794	6.794	6.034	0.293
**Block (offset) effects**	Any to any	0.646	-1.736	3.250	0.653	1.520
	Anti- to anti-FGMC	-3.781	-5.832	-1.749	-3.764	1.243
	Anti- to pro-FGMC	-4.186	-6.318	-2.128	-4.168	1.279
	Pro- to anti-FGMC	-4.164	-6.182	-1.960	-4.132	1.274
	Pro- to pro-FGMC	-5.273	-8.344	-2.536	-5.335	1.777
	Woman to woman	-5.089	-7.065	-3.218	-5.099	1.179
	Woman to man	-3.523	-5.355	-1.503	-3.536	1.177
	Man to woman	-5.307	-7.358	-3.407	-5.322	1.205
	Man to man	-4.085	-6.001	-2.151	-4.101	1.180
**Focal effects SD**		0.320	0.000	0.619	0.332	0.208
**Target effects SD**		0.910	0.668	1.157	0.917	0.151
**Dyadic effects SD**		0.190	0.000	0.485	0.229	0.180
**Focal-target effects rho**		-0.047	-0.630	0.565	-0.043	0.359
**Dyadic effects rho**		0.005	-0.653	0.663	0.009	0.403

Table S28 Full results from the STRAND kebele-zone 6 model exploring *social selection* in the latent *marriage-advice* networks

Variable		Median	LI	HI	Mean	SD
**Focal (out-degree) effects**	Age	-0.192	-0.692	0.308	-0.005	0.008
	Some primary vs. none	-0.293	-0.800	0.200	-0.252	0.260
	Complete primary vs. none	0.586	0.102	1.102	0.598	0.312
	Some secondary vs. none	0.035	-0.476	0.524	0.038	0.352
	Community role vs. none	-0.176	-0.674	0.326	-0.215	0.365
**Target (in-degree) effects**	Age	0.963	0.481	1.481	0.026	0.008
	Some primary vs. none	0.385	-0.115	0.885	0.352	0.282
	Complete primary vs. none	-0.460	-0.976	0.024	-0.730	0.477
	Some secondary vs. none	0.182	-0.324	0.676	0.220	0.378
	Community role vs. none	0.448	-0.067	0.933	0.521	0.358
**Dyadic (network overlap) effects**	Chatting	4.739	4.255	5.255	5.391	0.348
	Respect	3.582	3.084	4.084	4.050	0.345
	Money-borrowing	2.939	2.430	3.430	3.726	0.385
**Block (offset) effects**	Any to any	1.228	-1.220	3.775	1.228	1.512
	Anti- to anti-FGMC	-3.704	-5.757	-1.768	-3.720	1.220
	Anti- to pro-FGMC	-3.457	-5.454	-1.349	-3.470	1.248
	Pro- to anti-FGMC	-3.577	-5.578	-1.441	-3.585	1.263
	Pro- to pro-FGMC	-4.667	-7.951	-1.590	-4.777	1.930
	Woman to woman	-3.764	-5.688	-1.888	-3.771	1.159
	Woman to man	-3.697	-5.480	-1.660	-3.701	1.156
	Man to woman	-6.136	-8.152	-3.959	-6.144	1.295
	Man to man	-2.767	-4.669	-0.884	-2.769	1.156
**Focal effects SD**		0.167	0.000	0.395	0.194	0.145
**Target effects SD**		0.615	0.211	0.995	0.601	0.233
**Dyadic effects SD**		0.301	0.001	0.773	0.366	0.282
**Focal-target effects rho**		0.075	-0.559	0.744	0.062	0.398
**Dyadic effects rho**		0.041	-0.670	0.705	0.034	0.418

Table S29 Full results from the STRAND kebele-zone 7 model exploring *social selection* in the latent *marriage-advice* networks

Variable		Median	LI	HI	Mean	SD
**Focal (out-degree) effects**	Age	-0.375	-0.875	0.125	-0.006	0.005
	Some primary vs. none	-0.253	-0.804	0.196	-0.152	0.180
	Complete primary vs. none	-0.223	-0.714	0.286	-0.173	0.231
	Some secondary vs. none	-0.099	-0.613	0.387	-0.069	0.218
	Community role vs. none	0.062	-0.455	0.545	0.035	0.215
**Target (in-degree) effects**	Age	2.176	1.647	2.647	0.037	0.005
	Some primary vs. none	0.376	-0.123	0.877	0.237	0.196
	Complete primary vs. none	0.201	-0.321	0.679	0.179	0.273
	Some secondary vs. none	0.396	-0.114	0.886	0.318	0.242
	Community role vs. none	0.933	0.442	1.442	0.612	0.199
**Dyadic (network overlap) effects**	Chatting	7.616	7.146	8.146	7.124	0.287
	Respect	6.660	6.176	7.176	5.649	0.262
	Money-borrowing	7.237	6.747	7.747	5.691	0.240
**Block (offset) effects**	Any to any	1.464	-0.853	3.955	1.453	1.464
	Anti- to anti-FGMC	-4.262	-6.116	-2.260	-4.234	1.187
	Anti- to pro-FGMC	-5.084	-7.154	-3.058	-5.073	1.250
	Pro- to anti-FGMC	-4.267	-6.291	-2.256	-4.266	1.240
	Pro- to pro-FGMC	-2.690	-5.370	-0.201	-2.772	1.595
	Woman to woman	-4.399	-6.341	-2.600	-4.416	1.145
	Woman to man	-4.115	-5.928	-2.200	-4.129	1.145
	Man to woman	-4.917	-6.681	-2.917	-4.918	1.152
	Man to man	-4.013	-5.906	-2.199	-4.020	1.143
**Focal effects SD**		0.313	0.000	0.533	0.307	0.172
**Target effects SD**		0.652	0.495	0.820	0.653	0.100
**Dyadic effects SD**		0.293	0.000	0.705	0.341	0.250
**Focal-target effects rho**		0.037	-0.538	0.579	0.036	0.335
**Dyadic effects rho**		-0.132	-0.806	0.528	-0.119	0.410

Table S30 Full results from the STRAND kebele-zone 8 model exploring *social selection* in the latent *marriage-advice* networks

**Variable**		**Median**	**LI**	**HI**	**Mean**	**SD**
**Focal (out-degree) effects**	Age	-0.467	-1.000	0.000	-0.007	0.005
	Some primary vs. none	-0.062	-0.557	0.443	-0.035	0.169
	Complete primary vs. none	-0.167	-0.669	0.331	-0.139	0.250
	Some secondary vs. none	0.198	-0.296	0.704	0.130	0.199
	Community role vs. none	-0.152	-0.697	0.303	-0.165	0.304
**Target (in-degree) effects**	Age	2.111	1.611	2.611	0.038	0.005
	Some primary vs. none	0.319	-0.150	0.850	0.206	0.195
	Complete primary vs. none	0.167	-0.349	0.651	0.161	0.306
	Some secondary vs. none	0.001	-0.507	0.493	0.001	0.257
	Community role vs. none	0.507	-0.006	0.994	0.447	0.275
**Dyadic (network overlap) effects**	Chatting	9.313	8.800	9.800	7.470	0.246
	Respect	7.343	6.824	7.824	4.993	0.209
	Money-borrowing	8.632	8.160	9.160	6.598	0.233
**Block (offset) effects**	Any to any	0.757	-1.685	3.180	0.786	1.479
	Anti- to anti-FGMC	-3.881	-5.814	-1.981	-3.878	1.184
	Anti- to pro-FGMC	-4.580	-6.670	-2.701	-4.613	1.228
	Pro- to anti-FGMC	-3.659	-5.601	-1.596	-3.656	1.225
	Pro- to pro-FGMC	-5.372	-8.534	-2.369	-5.436	1.901
	Woman to woman	-5.177	-7.133	-3.296	-5.241	1.174
	Woman to man	-4.056	-5.987	-2.183	-4.072	1.174
	Man to woman	-5.339	-7.230	-3.426	-5.373	1.174
	Man to man	-3.955	-5.786	-1.986	-4.005	1.170
**Focal effects SD**		0.223	0.000	0.424	0.232	0.140
**Target effects SD**		0.804	0.639	0.966	0.807	0.103
**Dyadic effects SD**		0.225	0.000	0.568	0.267	0.212
**Focal-target effects rho**		0.269	-0.254	0.774	0.232	0.329
**Dyadic effects rho**		0.082	-0.602	0.735	0.068	0.417

Table S31 Full results from the STRAND kebele-zone 9 model exploring *social selection* in the latent *marriage-advice* networks

Variable		Median	LI	HI	Mean	SD
**Focal (out-degree) effects**	Age	0.500	-0.036	0.964	0.014	0.008
	Some primary vs. none	0.154	-0.317	0.683	0.156	0.313
	Complete primary vs. none	-0.163	-0.648	0.352	-0.268	0.465
	Some secondary vs. none	0.148	-0.368	0.632	0.198	0.409
	Community role vs. none	-0.358	-0.865	0.135	-0.586	0.491
**Target (in-degree) effects**	Age	1.206	0.706	1.706	0.042	0.010
	Some primary vs. none	0.003	-0.526	0.474	0.006	0.403
	Complete primary vs. none	-0.013	-0.471	0.529	-0.012	0.508
	Some secondary vs. none	-0.020	-0.522	0.478	-0.019	0.506
	Community role vs. none	-0.182	-0.684	0.316	-0.266	0.430
**Dyadic (network overlap) effects**	Chatting	3.764	3.263	4.263	4.929	0.407
	Respect	2.800	2.298	3.298	3.327	0.363
	Money-borrowing	2.104	1.628	2.628	2.774	0.397
**Block (offset) effects**	Any to any	0.963	-1.509	3.663	0.981	1.576
	Anti- to anti-FGMC	-3.036	-5.301	-0.823	-3.030	1.369
	Anti- to pro-FGMC	-6.873	-10.780	-3.349	-6.966	2.273
	Pro- to anti-FGMC	-3.583	-5.796	-1.103	-3.582	1.439
	Pro- to pro-FGMC	-1.203	-4.118	1.707	-1.199	1.788
	Woman to woman	-5.153	-7.170	-3.125	-5.139	1.223
	Woman to man	-3.041	-4.946	-0.988	-3.020	1.203
	Man to woman	-4.605	-6.600	-2.582	-4.607	1.223
	Man to man	-3.166	-5.084	-1.116	-3.153	1.205
**Focal effects SD**		0.176	0.000	0.435	0.210	0.159
**Target effects SD**		0.658	0.294	1.076	0.668	0.236
**Dyadic effects SD**		0.387	0.001	0.983	0.461	0.352
**Focal-target effects rho**		0.108	-0.558	0.729	0.090	0.399
**Dyadic effects rho**		0.091	-0.610	0.750	0.073	0.418

Table S32 Demographic characteristics by kebele-zone for the full sample and the network respondents subsample

			Kebele-zone								
			**1**	**2**	**3**	**4**	**5**	**6**	**7**	**8**	**9**
**n**	Full sample		1031	400	316	322	633	305	820	1122	214
	Network subsample		509	200	159	158	301	154	411	551	102
			Median (IQR)								
**Age (years)**	Full sample		30 (22)	28 (23.5)	30 (24.25)	30 (29.75)	29 (27)	30 (23)	32 (26)	30 (22)	35 (24)
	Network subsample		32 (20)	30.5 (23)	32 (24.5)	35 (30)	35 (28)	35 (17.75)	35 (25)	34 (21.5)	35 (24.5)
			Percentage								
**Pro-FGMC**	Full sample		12.124	9.500	8.861	7.764	7.109	4.262	2.683	2.317	1.412
	Network subsample		11.395	11	10.692	6.962	8.306	4.545	2.19	2.359	2.941
**Male**	Full sample		48.788	53.081	47.196	49.684	49.287	49.250	47.205	48.852	51.341
	Network subsample		48.527	50	50.314	46.203	51.827	48.052	51.582	49.002	46.078
**Highest education**	None	Full sample	24.442	27.000	27.215	24.534	23.697	27.213	21.707	22.549	27.103
		Network subsample	25.344	30	28.931	24.051	27.243	28.571	23.601	24.501	27.451
	Some primary	Full sample	48.109	48.750	49.051	50.311	50.237	45.246	42.805	44.652	46.729
		Network subsample	47.151	49.5	50.314	48.734	50.166	47.403	42.822	44.283	42.157
	Completed primary	Full sample	9.893	10.500	12.025	12.422	10.742	12.459	12.805	11.854	10.280
		Network subsample	9.234	7.5	10.063	13.924	9.635	11.688	14.119	9.982	12.745
	Some secondary or beyond	Full sample	17.556	13.75	11.709	12.733	15.324	15.082	22.683	20.945	15.888
		Network subsample	18.271	13	10.692	13.291	12.957	12.338	19.465	21.234	17.647
**Community role**	None	Full sample	92.629	93.250	89.241	93.478	93.049	91.475	89.634	94.474	91.589
		Network subsample	92.338	92.5	88.679	92.405	91.362	91.558	88.564	95.1	92.157
	Role	Full sample	7.371	6.750	9.810	6.522	6.951	8.525	10.366	5.526	8.411
		Network subsample	7.662	7.5	11.321	7.595	8.638	8.442	11.436	4.9	7.843

Table S33 Within-kebele-zone advice out and advice in nominations used in the STRAND models (n = number of nominators)

	1	2	3	4	5	6	7	8	9
n	509	200	159	158	301	154	411	551	102
Advice out	744	284	248	223	398	240	701	764	167
Advice in	717	245	238	213	376	217	612	645	131

Figures

Figure S1 Directed acyclic graph (DAG)

Plot of the directed acyclic graph (DAG) of assumed causal pathways between measures thought important in the process of an individual arriving at their personal preference regarding FGMC. Here the exposure variable is the presense of a pro-FGMC alter within an individual's network (FGMC_POS_TIE), reflecting the contagion parameter in our ALAAMs), and the outcome variable is an individual's FGMC-preference (FGMC). Based on these assumed relationships between variables, the minimally sufficient adjustment set in the ALAAMs is age, education (EDU), gender, zone, in-degree, and out-degree. Further abbreviations: EMP_W – empirical expectations regarding women, EMP_M – empirical expectations regarding men, NORM – normative expectations. Graph created using the web-based app version of dagitty (Textor, Hardt, and Knüppel 2011), see dagitty.net for more information regarding colour coding.

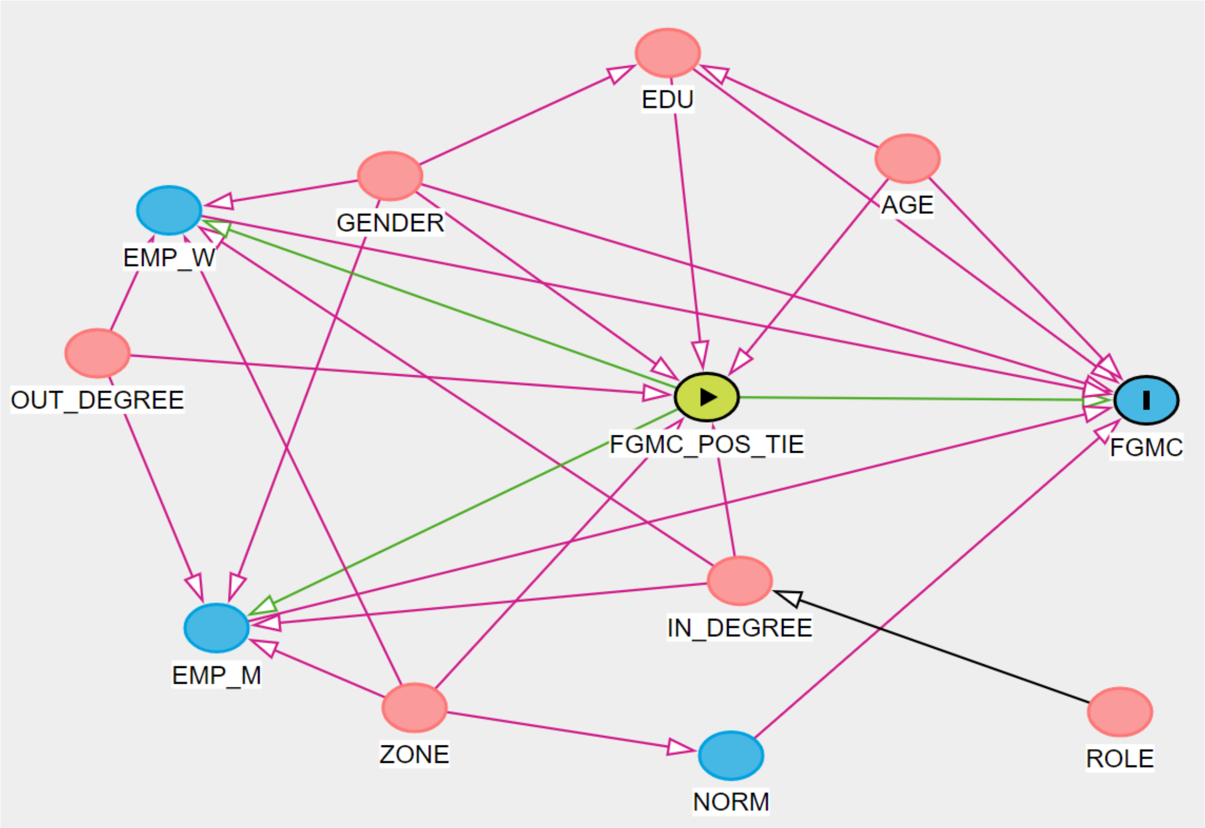

Figure S2 The distribution of FGMC preference dependent on A) age, B) gender, C) highest education level, and D) community role

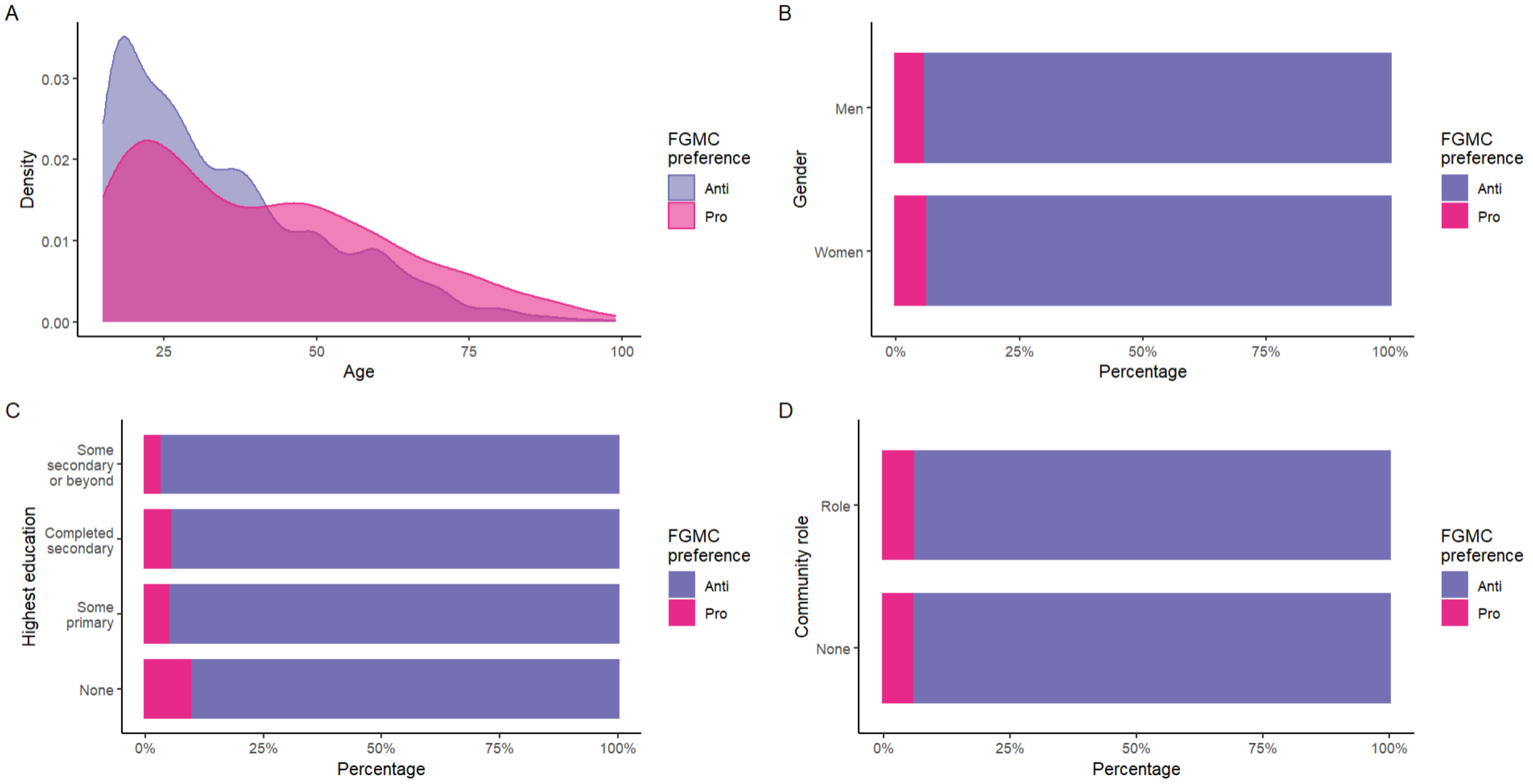

Figure S3 Comparing empirical expectations between the two largest kebele-zones

The distribution of *empirical expectations* regarding the prevalence of support for FGMC among men (A-B) and women (C-D) in kebele-zone's 1 (A, C) and 8 (B, D), i.e. the two largest kebele-zones, dependent on whether the respondent reported holding pro- or anti-FGMC preference themself. Zone 1 n = 1031, pro-FGMC = 12.1%; zone 8 n = 1122, pro-FGMC = 2.3%.

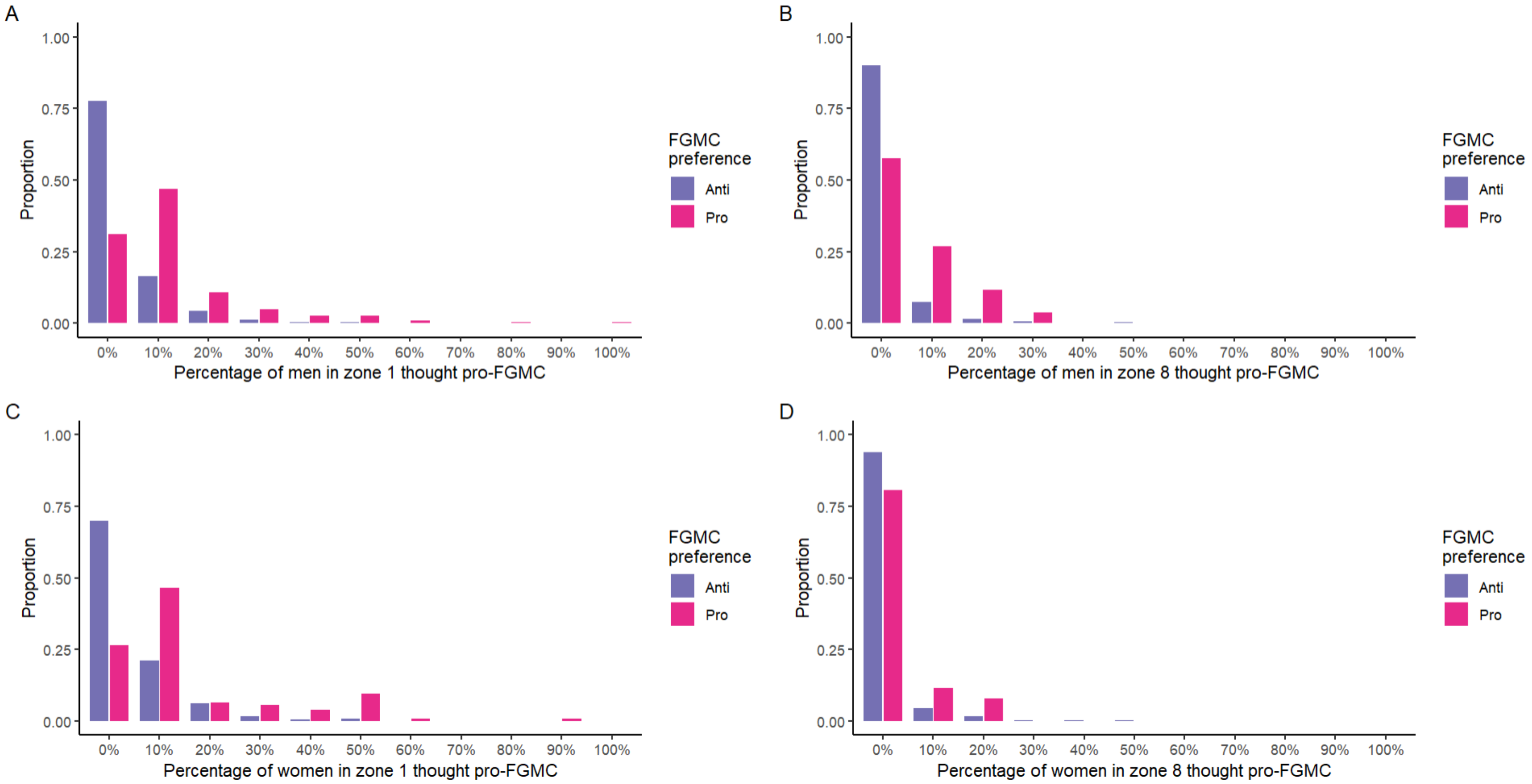

Table S4 The proportional distribution of relationship types among network ties by FGMC preference

The proportional distribution of nominations in response to name generators – A) *chatting*, B) *respect*, C) *borrow out*, D) *borrow in*, E) *advice out*, and F) *advice in* – by relationship type, dependent on whether the nominator reported holding pro- (pink, upper bar) or anti-FGMC (purple, lower bar) preference.

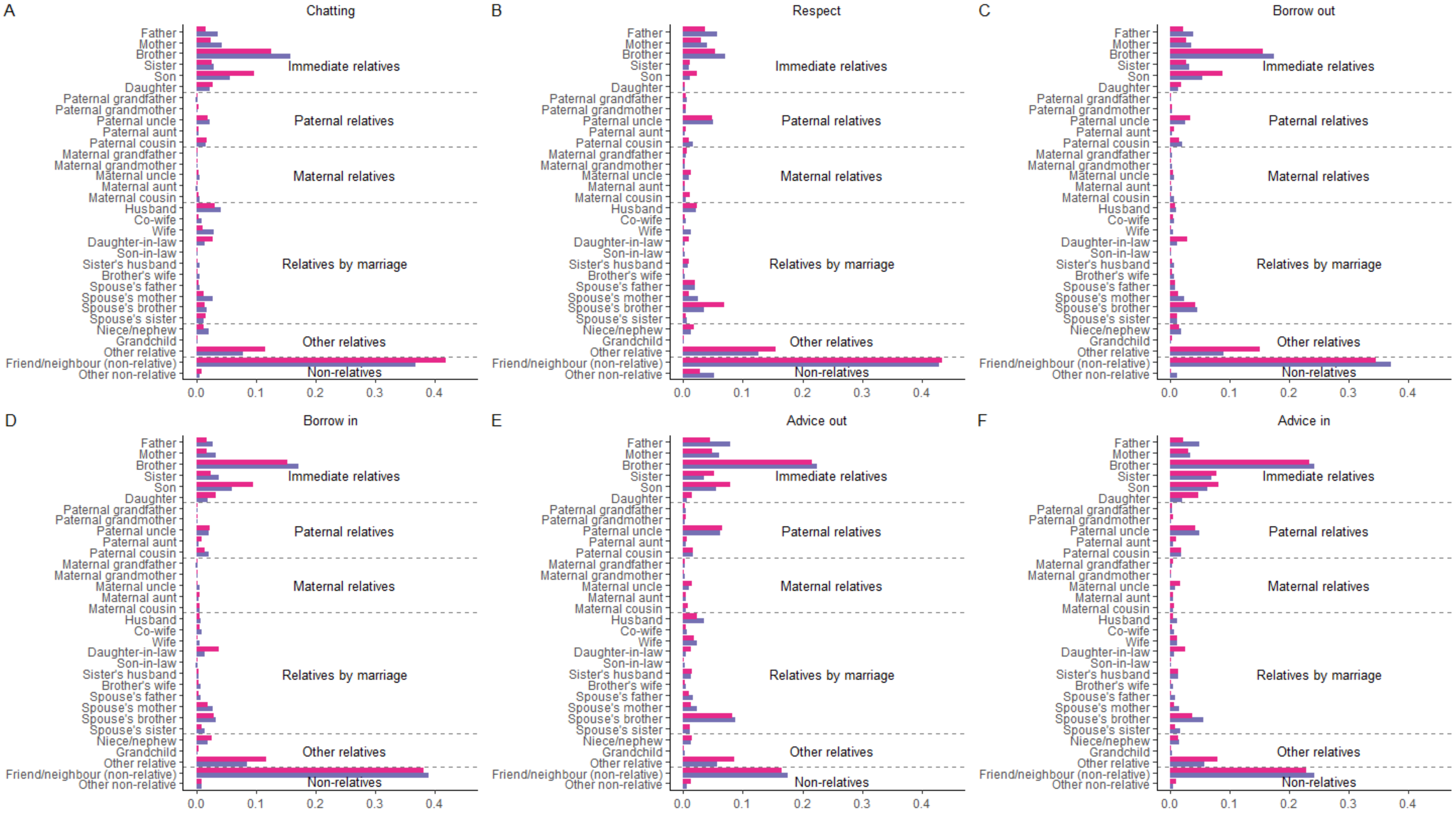

Figure S5 Plot of *respect* ties

Plot of *respect* ties between 5163 respondents to the *Norms and Networks Survey* as a whole, reported by the 2545 respondents asked for network data. Circles reflect women and triangles reflect men. Plotted using the Fruchterman-Reingold algorithm, ties can be seen to cluster by zone, with only limited ties between individuals in different zones. Not all respondents to the survey were named as respected individuals (n=1862) (including 40 network respondents who both named an entirely unknown network and were not themselves nominated), hence the presence of isolated nodes at the outer rim.

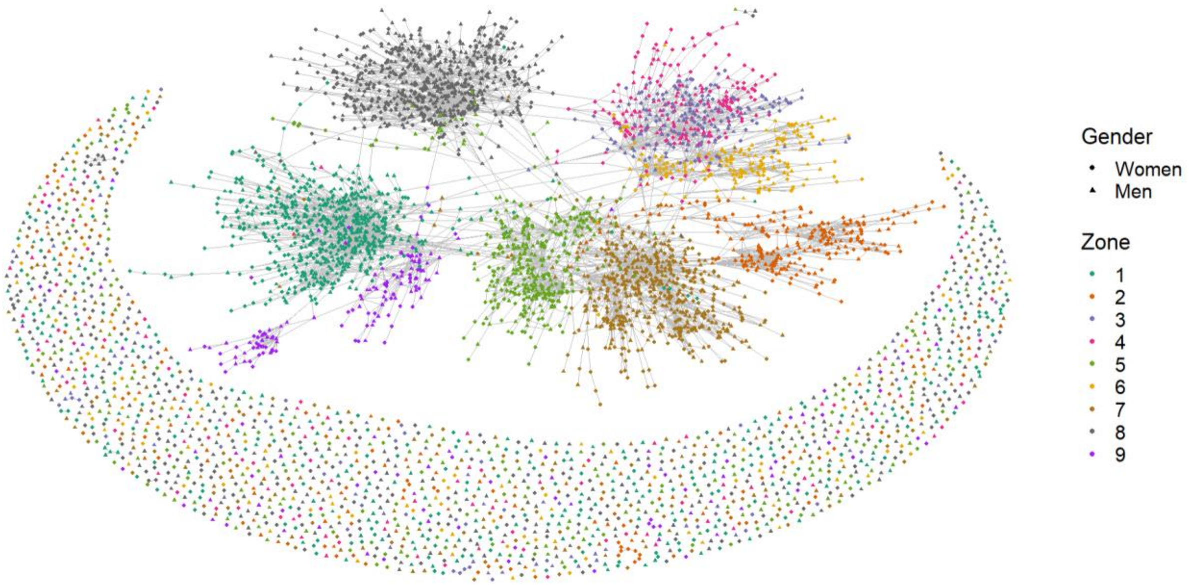

Figure S6 Plot of estimated latent *money-borrowing* ties

Plot of estimated latent *money-borrowing* ties between 5163 respondents to the *Norms and Networks Survey* as a whole, reported by the 2545 respondents asked for network data. Circles reflect women and triangles reflect men. Plotted using the Fruchterman-Reingold algorithm, ties can be seen to cluster by zone, with only limited ties between individuals in different zones. As not all respondents to the survey were named in response to the two name generators they are estimated to not be connected to other individuals in the network, hence the presence of isolated nodes (n=1201) at the outer rim. For legend see Figure S5.

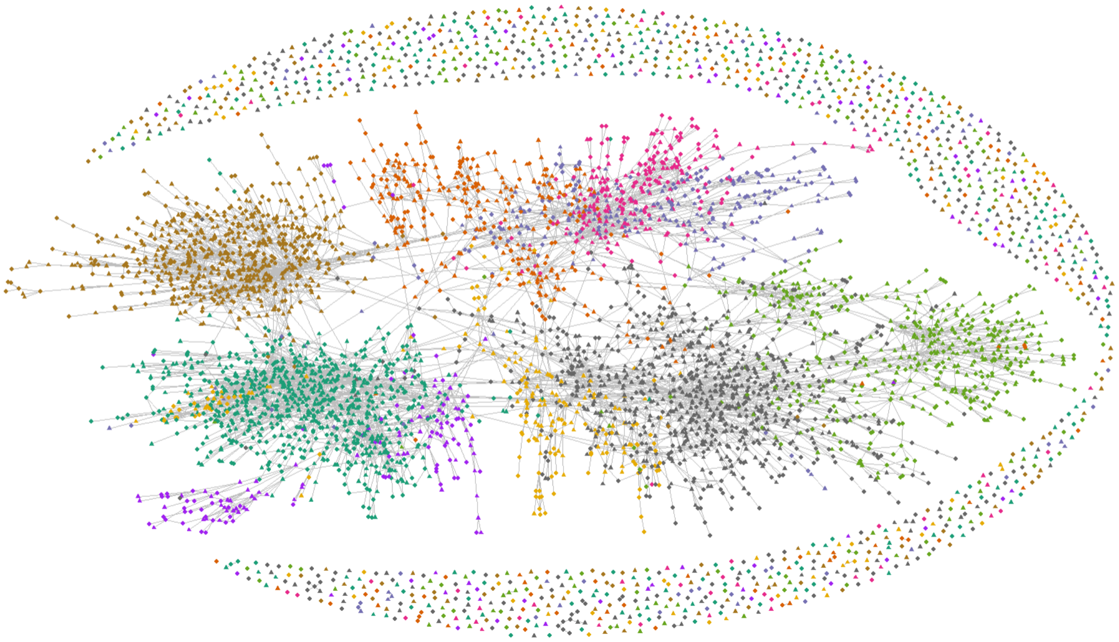

Figure S7 Plot of estimated latent *marriage-advice* ties

Plot of estimated latent *marriage-advice* ties between 5163 respondents to the *Norms and Networks Survey* as a whole, reported by the 2545 respondents asked for network data. Circles reflect women and triangles reflect men. Plotted using the Fruchterman-Reingold algorithm, ties can be seen to cluster by zone, with only limited ties between individuals in different zones. As not all respondents to the survey were named in response to the two name generators they are estimated to not be connected to other individuals in the network, hence the presence of isolated nodes (n=1346) at the outer rim. For legend see Figure S5.

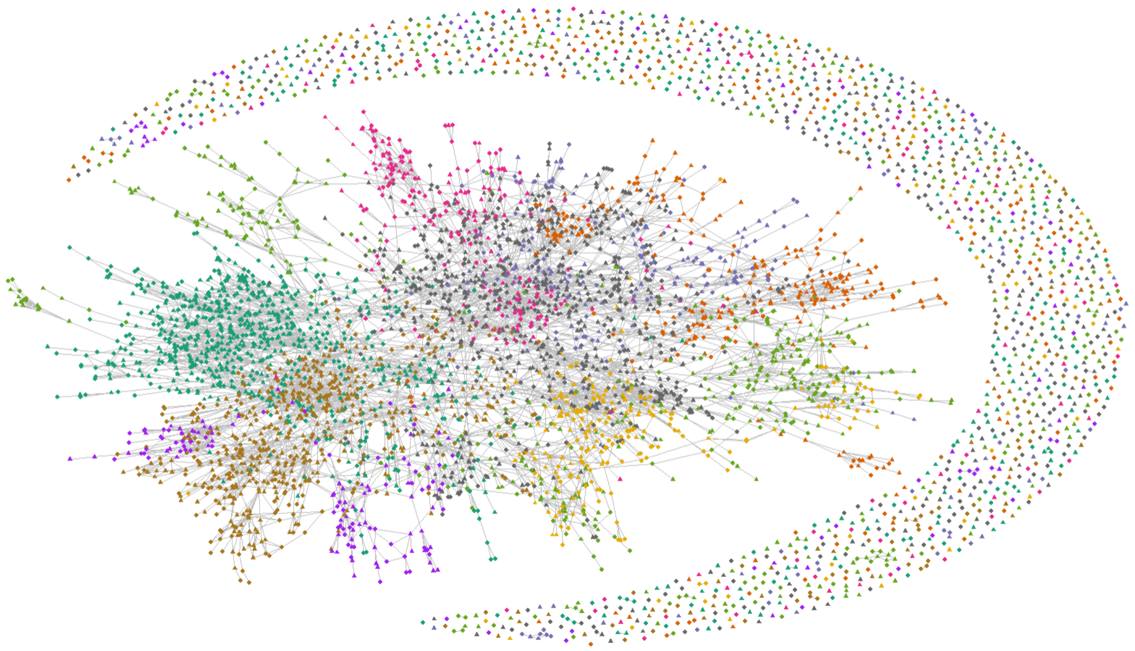

Figure S8 Social influence signals across networks. Plotted are the posterior distributions for the estimates for social contagion from the best fitting ALAAM models assessing either all, kin-only, or non-kin-only alters within the A) *chatting,* B) *respect*, and C) latent *money-borrowing* networks.

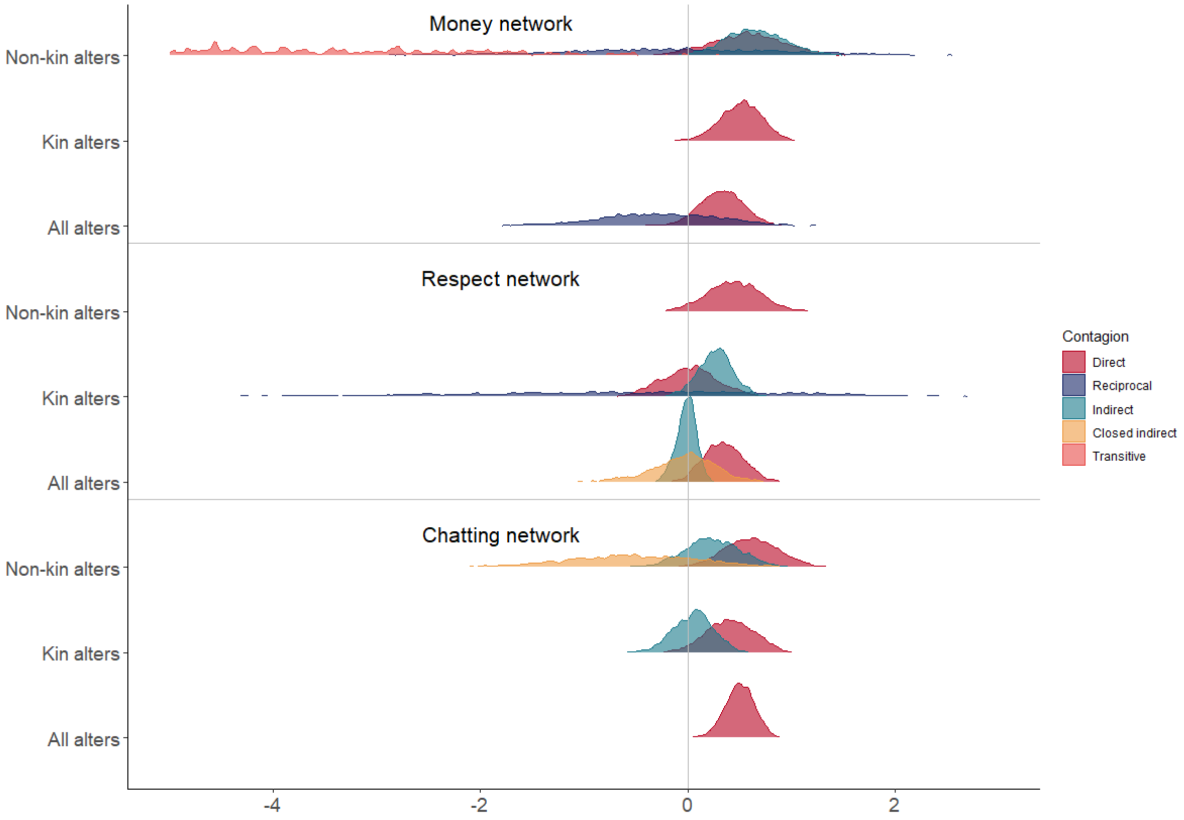

Figure S9 Posterior distributions for the *money-borrowing* model only using network reporters

Plotted are the posterior distributions for the estimates for social contagion from ALAAM models assessing either all, kin-only, or non-kin only alters within latent *money-borrowing* networks when using the subsample of only respondents who also reported their networks. Estimates above zero signal a positive contagion effect. The numbers listed give the proportion of the posterior above the null (i.e. zero).

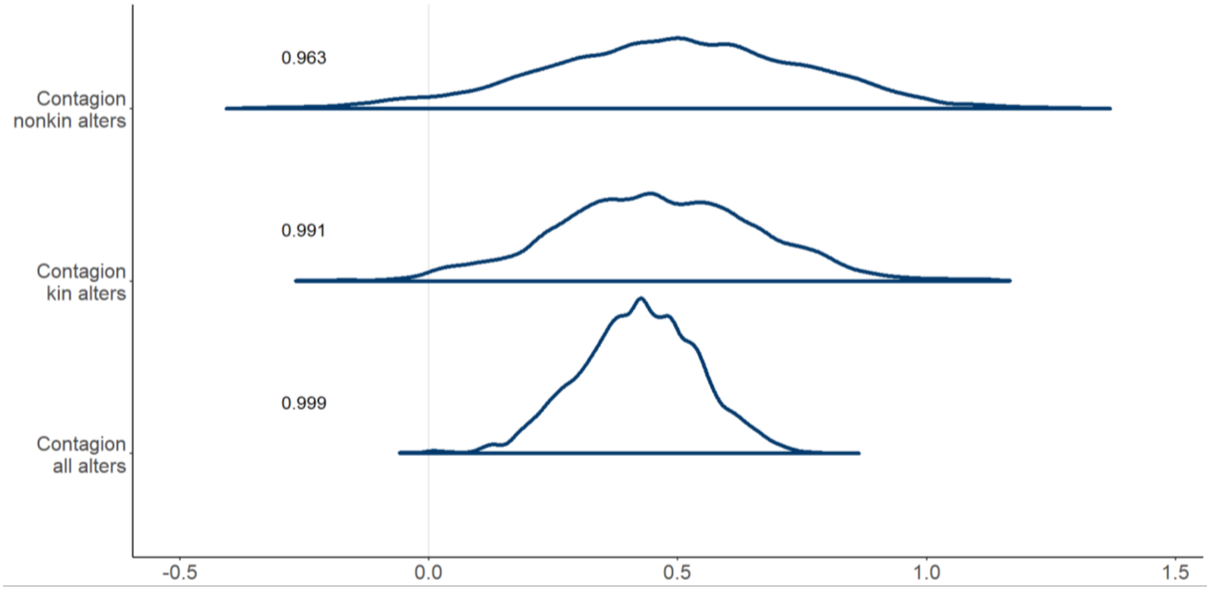

Figure S10 *Social selection* model results – FGMC preference block effects

A) Plot of estimates in log-odds of a tie offset against a global intercept term, from which the contrasts in B-D were calculated. B-D) Plot of contrast coefficients for estimates of the probability of a given dyad type compared to the reference type – B)'pro to pro', C) 'anti to anti', and D) 'pro to anti' *marriage-advice* ties. Non-overlapping estimates can generally be considered to be consistently different, while contrasts quantify the difference; as such there is negligible evidence of differential clustering by FGMC preference. The point estimate reflects the median and the error bars the highest posterior density interval (HPDI) from the posterior distribution. For the number of advice out and advice in nominations contributing to the latent networks per kebele-zone see SI Table S33.

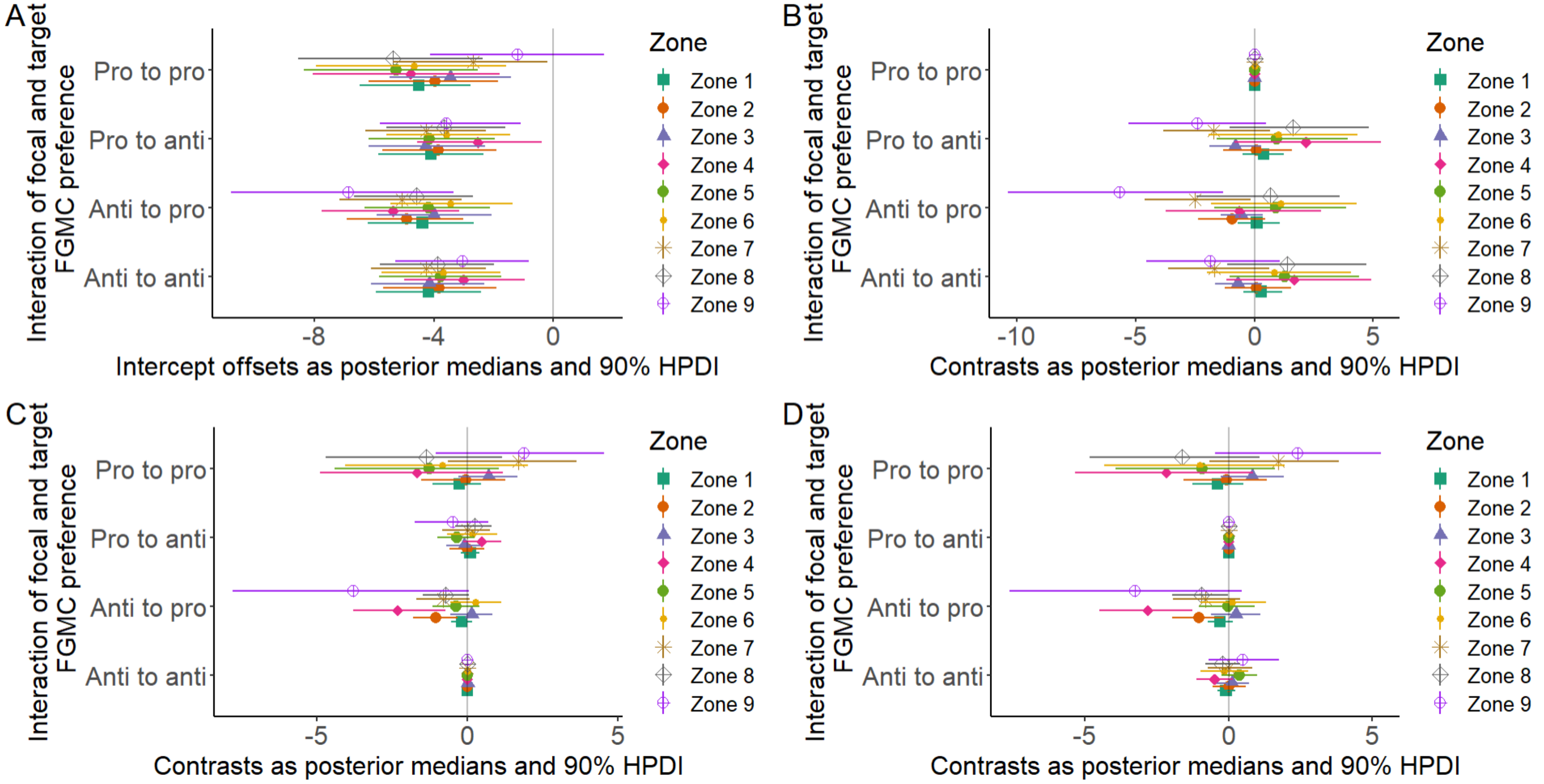

Figure S11 Reciprocity and variation in *marriage-advice* ties

A) Plot of the correlation parameter, ρ, for dyadic effects indicates the potential for advice nominations to be reciprocal, after adjusting for the individual-level variation in the probability of sending and receiving ties. The ρ for focal-target effects indicates the strength of the association between the individual-level probability of sending and receiving ties. Perhaps unsurprisingly, there is little evidence of advice ties being reciprocal and seeking advice does not correlate with being sought for advice. B) The standard deviation parameters, σ, indicate the level of variation in dyadic, target/receiver, and focal/sender random effects. The point estimate reflects the median and the error bars the highest posterior density interval (HPDI) from the posterior distribution. For the number of advice out and advice in nominations contributing to the latent networks per kebele-zone see SI Table S33.

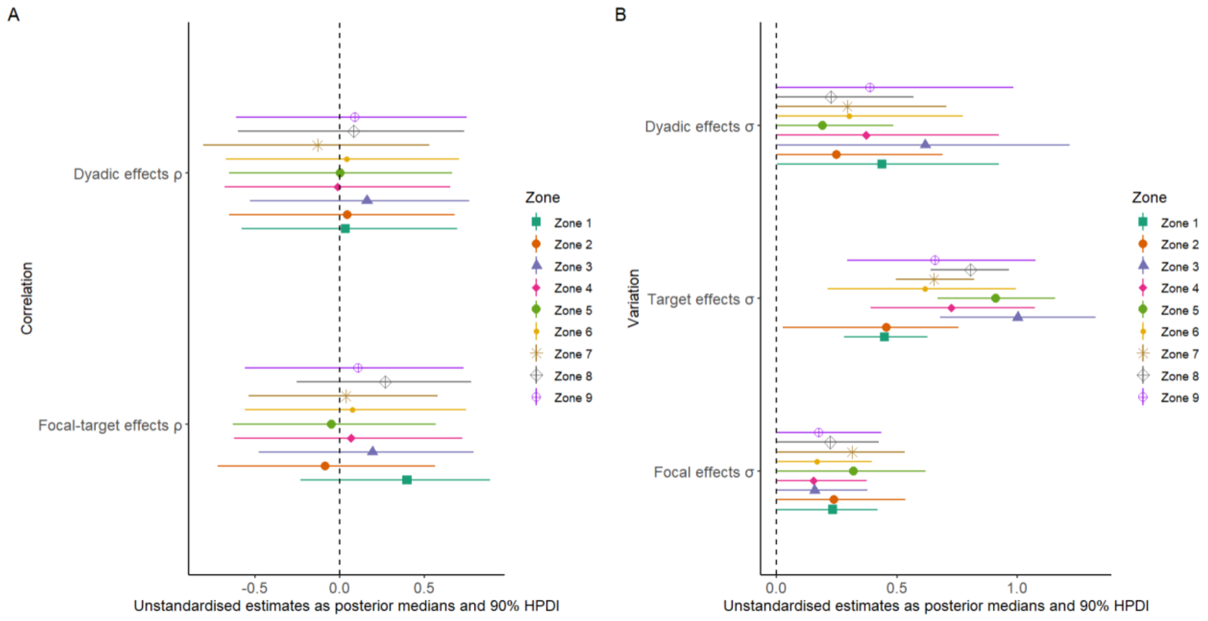

Figure S12 *Social selection* model results – focal (out-degree) and target (in-degree) effects

A) Plot of estimates for individual-level parameters predicting the likelihood of seeking advice across zones. No parameter consistently positively or negatively predicted sending an advice tie across zones. B) Plot of estimates for individual-level parameters predicting the likelihood of being sought for advice across zones. Only age was estimated to have a consistent effect across zones, with older individuals more likely to named as sources of advice. The point estimate reflects the median and the error bars the highest posterior density interval (HPDI) from the posterior distribution. For the number of advice out and advice in nominations contributing to the latent networks per kebele-zone see SI Table S33.

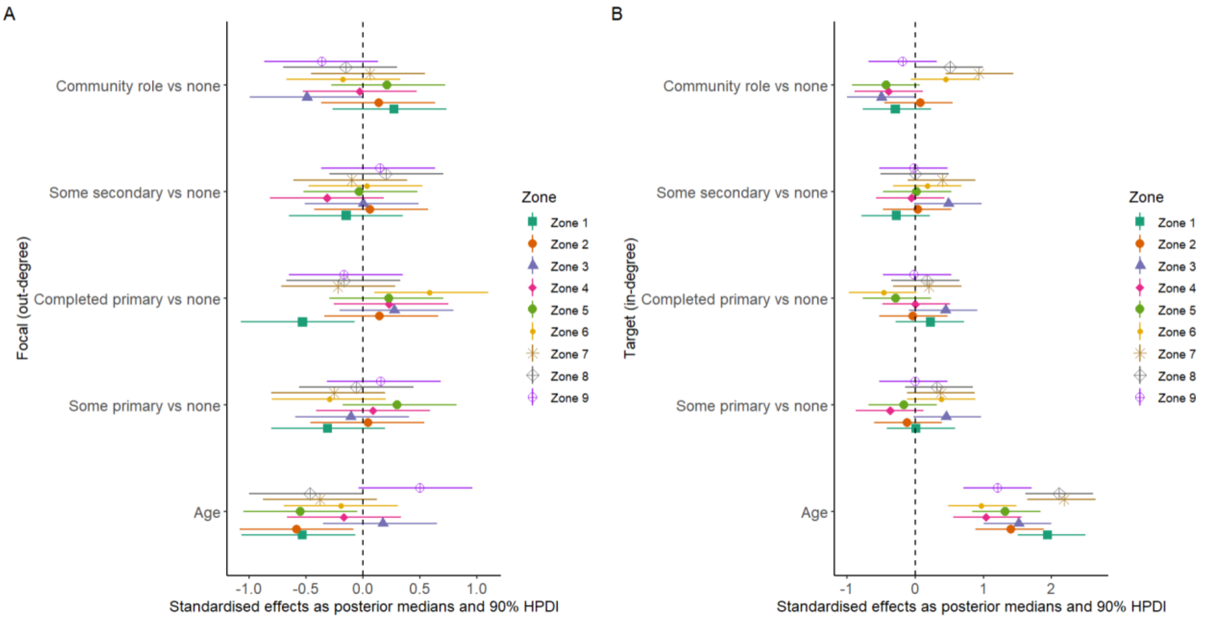

Figure S13 *Social selection* model – dyadic effects

Plot of the standardised estimates for the effects of dyad-level parameters on the likelihood of an advice tie existing between any two members of the network. Results indicate that sharing a tie in the *respect*, *chatting*, and *money-borrowing* networks increased the likelihood of an advice tie in all zones. The point estimate reflects the median and the error bars the highest posterior density interval (HPDI) from the posterior distribution. For the number of advice out and advice in nominations contributing to the latent networks per kebele-zone see SI Table S33.

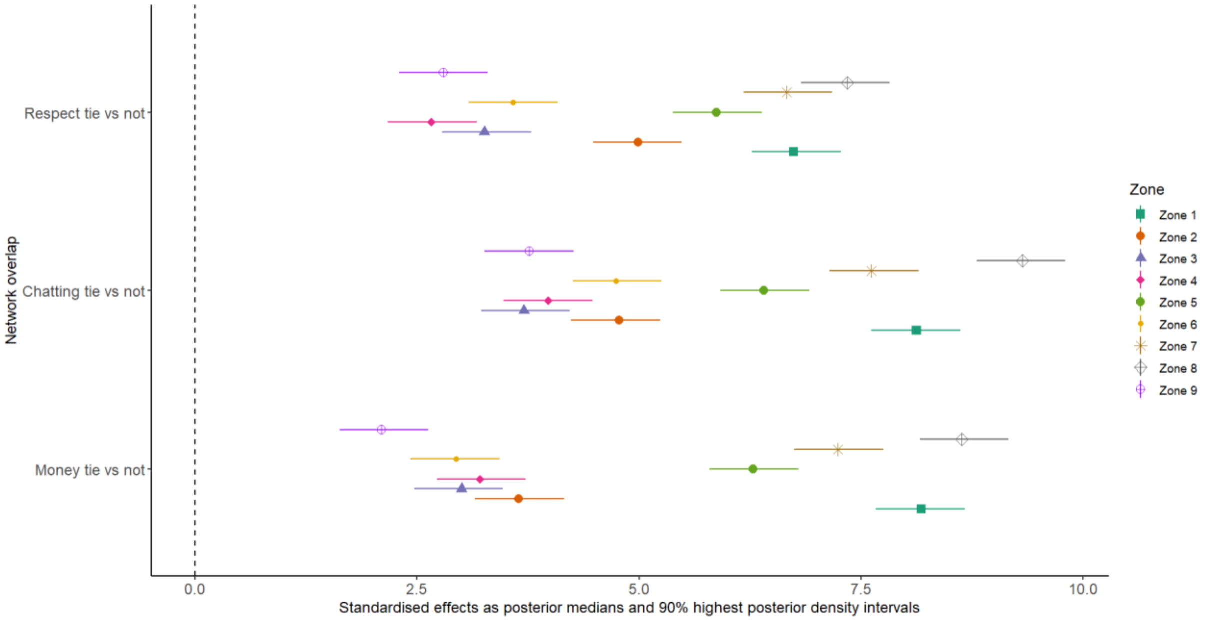

Figure S14 *Social selection* model results – gender block effects

A) Plot of contrasts for estimates of the likelihood of a given dyad type compared to the reference type (woman to woman *marriage-advice* ties), broadly indicating a bias towards seeking advice from men. B) Plot of estimates in log-odds of a tie offset against a global intercept term, from which the contrasts in A are calculated. Non-overlapping estimates can generally be considered to be consistently different, while contrasts quantify the difference; as such the gender bias seen in A is only weakly supported. The point estimate reflects the median and the error bars the highest posterior density interval (HPDI) from the posterior distribution. For the number of advice out and advice in nominations contributing to the latent networks per kebele-zone see SI Table S33.

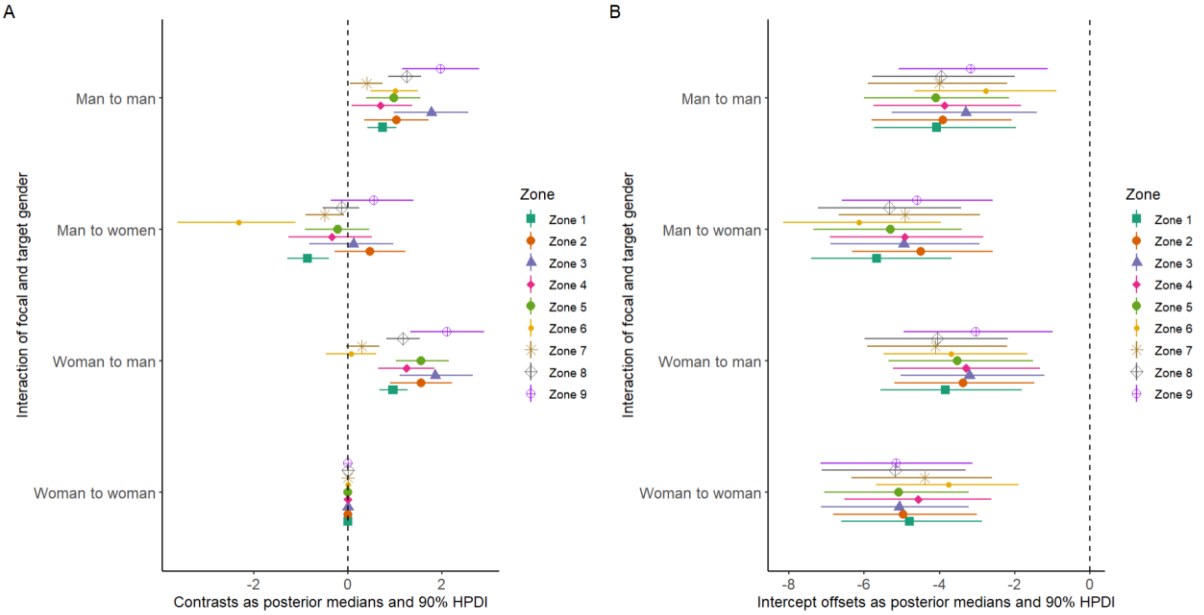

References

Koskinen, J. and Daraganova, G., 2022. Bayesian analysis of social influence. *Journal of the Royal Statistical Society Series A: Statistics in Society*, *185*(4), pp.1855-1881.

Textor, J., Hardt, J. and Knüppel, S., 2011. DAGitty: a graphical tool for analyzing causal diagrams. *Epidemiology*, *22*(5), p.745.
