# Supplementary material for: Social clustering of preference for female genital mutilation/cutting in south-central Ethiopia
Source: Nat Hum Behav. 2025 Jun 10;9(9):1802–14. doi: 10.1038/s41562-025-02236-z (PMC12454122; doi:10.1038/s41562-025-02236-z)
Supplement: Supplementary file 2 — Reporting Summary [file 41562_2025_2236_MOESM2_ESM.pdf]

nature portfolio

Corresponding author(s): Mhairi Gibson NATHUMBEHAV-24051775B

Last updated by author(s): 2025/04/25

Reporting Summary

Nature Portfolio wishes to improve the reproducibility of the work that we publish. This form provides structure for consistency and transparency in reporting. For further information on Nature Portfolio policies, see our Editorial Policies and the Editorial Policy Checklist.

Statistics

For all statistical analyses, confirm that the following items are present in the figure legend, table legend, main text, or Methods section.

n/a	Confirmed	
☐	☒	The exact sample size (*n*) for each experimental group/condition, given as a discrete number and unit of measurement
☐	☒	A statement on whether measurements were taken from distinct samples or whether the same sample was measured repeatedly
☐	☒	The statistical test(s) used AND whether they are one- or two-sided<br>*Only common tests should be described solely by name; describe more complex techniques in the Methods section.*
☐	☒	A description of all covariates tested
☐	☒	A description of any assumptions or corrections, such as tests of normality and adjustment for multiple comparisons
☐	☒	A full description of the statistical parameters including central tendency (e.g. means) or other basic estimates (e.g. regression coefficient) AND variation (e.g. standard deviation) or associated estimates of uncertainty (e.g. confidence intervals)
☒	☐	For null hypothesis testing, the test statistic (e.g. *F*, *t*, *r*) with confidence intervals, effect sizes, degrees of freedom and *P* value noted<br>*Give P values as exact values whenever suitable.*
☐	☒	For Bayesian analysis, information on the choice of priors and Markov chain Monte Carlo settings
☐	☒	For hierarchical and complex designs, identification of the appropriate level for tests and full reporting of outcomes
☒	☐	Estimates of effect sizes (e.g. Cohen's *d*, Pearson's *r*), indicating how they were calculated

Our web collection on statistics for biologists contains articles on many of the points above.

Software and code

Policy information about availability of computer code

Data collection	No software used, papser surveys only.
Data analysis	Data analysis was conducted using R Studio version 4.1.3; social selection modelling was conducted using the package STRAND version 0.0.0.9000; social influence modelling was conducted with the BayesALAAM function, part of MultivarALAAMalt.R available on GitHub and downloaded on 23/08/2023. All code used is available on the Open Science Framework at https://osf.io/765vg/.

For manuscripts utilizing custom algorithms or software that are central to the research but not yet described in published literature, software must be made available to editors and reviewers. We strongly encourage code deposition in a community repository (e.g. GitHub). See the Nature Portfolio guidelines for submitting code & software for further information.

Data

Policy information about availability of data

All manuscripts must include a data availability statement. This statement should provide the following information, where applicable:

- Accession codes, unique identifiers, or web links for publicly available datasets
- A description of any restrictions on data availability
- For clinical datasets or third party data, please ensure that the statement adheres to our policy

The data is available on the Open Science Framework at https://osf.io/765vg/.

Research involving human participants, their data, or biological material

Policy information about studies with human participants or human data. See also policy information about sex, gender (identity/presentation), and sexual orientation and race, ethnicity and racism.

Reporting on sex and gender	Throughout the manuscript we refer to the gender of survey respondents, as this is the most accurate terminology by academic standards. This information was inferred by field assistants conducting interviews, so as not to cause offense to respondents (a risk in this study context). However, the study community socially recognises only two genders, drawing no distinction between the concepts of sex and gender, and local field assistants originally coding the data recorded a variable 'sex' with options 'male' or 'female'. For the sake of transparency, we have retained this wording, altering the terminology during the model coding and signposting where this occurs in the code notation.<br>The sample is composed of 49.8% men and 50.2% women.<br>As gender likely plays a role in the development of thoughts and feelings regarding female genital mutilation/cutting, various outcomes of interest are reported disaggregated by gender and it is included as a parameter in our models.
Reporting on race, ethnicity, or other socially relevant groupings	The study communities were selected for the ethnic homogeneity (Arsi Oromo), which is of relevance when a cultural behaviour such as female genital mutilation/cutting. During the review process, information regarding self-reported religious affiliation was added, serving only to reinforce the relative homogeneity of the sample: Muslim 94.1%, Orthodox Christian 5.8%, other 0.1%. Neither ethnicity or religion are included within our analyses.<br>Respondents are grouped by self-reported educational attainment and community role.<br>We use directed acyclic graphs to determine the variables to include in our models to minimise confounding.
Population characteristics	N = 5163<br>Gender: men 49.8%, women 50.2%.<br>Age: median 30 years, IQR 25 years, range 15 - 99 years (note age is a self-reported approximation in this context).<br>Education: none 24.2%, some primary 46.8%, completed primary 11.4%, some secondary or beyond 17.7%.<br>Community role: none 92.5%, role 7.6%.<br>FGMC preference: pro-FGMC 6.3%, anti-FGMC 93.7%.
Recruitment	Every household in the study area, identified using government lists, was initially censused; household's were then visited again and invited to take part in the main study
Ethics oversight	University of Bristol (UK) and Addis Ababa University (Ethiopia).

Note that full information on the approval of the study protocol must also be provided in the manuscript.

Field-specific reporting

Please select the one below that is the best fit for your research. If you are not sure, read the appropriate sections before making your selection.

☐ Life sciences ☒ Behavioural & social sciences ☐ Ecological, evolutionary & environmental sciences

For a reference copy of the document with all sections, see nature.com/documents/nr-reporting-summary-flat.pdf

Behavioural & social sciences study design

All studies must disclose on these points even when the disclosure is negative.

Study description	The study is a quantitative, cross-sectional, observational study.
Research sample	The research sample approximates the entire adult population (individuals aged 15 years or over) of 9 neighbouring kebele-zones (N = 5163), thus is representative of these communities. Kebele-zones were selected due to their high ethnic homogeneity, being predominantly ethnically Arsi Oromo, known to practice female genital mutilation/cutting (FGMC), and geographical contiguity.
Sampling strategy	The entire population was targeted for inclusion
Data collection	Data collection took place between 2021 and 2022. Data was collected from nine neighbouring administrative kebele-zones (rural villages), distributed across three neighbouring Kebeles (sub districts) in South Central Ethiopia. Kebele-zones were selected due to their being predominantly ethnically Arsi Oromo, known to practice female genital mutilation (FGMC), and geographical contiguity. Data was recorded using pen and paper. Surveys were conducted in Afan Oromo by research assistants trained in demographic field survey methods, recruited and trained by EG at Addis Ababa University.
Timing	Surveys were undertaken 2021-2022.
Data exclusions	Data from 18 interviews were excluded from analyses: 2 cases were excluded due to irreconcilable recording errors and 16 exclusions were made to remove one set of responses where individuals were interviewed twice.

Non-participation	Our household census recorded 5578 adults residing within our study population, of whom 5165 (92.6%) took part in the study. Reasons for non-participation were not systematically documented but include uncontactability.
Randomization	Participants were not allocated into experimental groups.

Reporting for specific materials, systems and methods

We require information from authors about some types of materials, experimental systems and methods used in many studies. Here, indicate whether each material, system or method listed is relevant to your study. If you are not sure if a list item applies to your research, read the appropriate section before selecting a response.

Materials & experimental systems

n/a	Involved in the study	
☒	☐	Antibodies
☒	☐	Eukaryotic cell lines
☒	☐	Palaeontology and archaeology
☒	☐	Animals and other organisms
☒	☐	Clinical data
☒	☐	Dual use research of concern
☒	☐	Plants

Methods

n/a	Involved in the study	
☒	☐	ChIP-seq
☒	☐	Flow cytometry
☒	☐	MRI-based neuroimaging

Plants

Seed stocks	*Report on the source of all seed stocks or other plant material used. If applicable, state the seed stock centre and catalogue number. If plant specimens were collected from the field, describe the collection location, date and sampling procedures.*
Novel plant genotypes	*Describe the methods by which all novel plant genotypes were produced. This includes those generated by transgenic approaches, gene editing, chemical/radiation-based mutagenesis and hybridization. For transgenic lines, describe the transformation method, the number of independent lines analyzed and the generation upon which experiments were performed. For gene-edited lines, describe the editor used, the endogenous sequence targeted for editing, the targeting guide RNA sequence (if applicable) and how the editor was applied.*
Authentication	*Describe any authentication procedures for each seed stock used or novel genotype generated. Describe any experiments used to assess the effect of a mutation and, where applicable, how potential secondary effects (e.g. second site T-DNA insertions, mosiacism, off-target gene editing) were examined.*
